# Respiratory Biomarkers: An Opportunity for Patient‐Centric Microsampling Approach for Treatment Optimization

**DOI:** 10.1111/cts.70600

**Published:** 2026-06-01

**Authors:** Ahmar Khan, Jacqueline L. Stair, Daniel Baker, Neil Spooner, Darragh Murnane

**Affiliations:** ^1^ Department of Medicine, Applied and Clinical Sciences, School of Health, Medicine, and Life Sciences University of Hertfordshire Hatfield Hertfordshire UK; ^2^ Spooner Bioanalytical Solutions Hertford Hertfordshire UK; ^3^ Patient Centric Sampling Interest Group CIC Ware Herts UK

**Keywords:** clinical markers, inter‐patient variability, patient‐centric sampling, personalized medicine, pulmonary disease

## Abstract

The clinical heterogeneity of respiratory disorders emphasizes the need for personalized approaches to prevention, diagnosis, and therapy. One approach to enhanced personalization of therapy is the identification of biomarkers for disease phenotype, severity, progression, and response to therapy. To date, several biomarkers have been developed for patients with respiratory diseases, and increasingly, blood‐derived protein and peptide biomarkers are under investigation for their potential to achieve better control over respiratory diseases by stratifying patients for specific treatment regimens. However, identifying and quantifying these biomarkers in a routine setup is challenging because of the invasiveness involved in sample collection, the need for in‐person clinical visits, and the need for skilled phlebotomy. Consequently, clinical biomarker measurements represent a single‐time point indication of health on the day of attendance at a clinic and not necessarily an accurate longitudinal representation of the patient's condition. Micro‐sampling techniques have developed the concept of patient‐centric sampling with minimal invasiveness, with the goal of the patient taking their own blood sample in micro volumes that are sent for analysis in a centralized laboratory, all from the comfort of their home. While novel micro‐sampling techniques have demonstrated promising results in quantifying drugs and proteins from blood, there is limited exploration within the domain of respiratory diseases' biomarkers. Hence, this article provides insights into novel respiratory biomarkers and a future that exploits micro‐sampling tools in care pathways to optimize and individualize therapy for patients.

## Graphical abstract

Overview of patient centric microsampling approach for monitoring respiratory biomarkers for treatment optimization. Image created in BioRender. Khan, A. (2026) https://BioRender.com/17jm5cs.

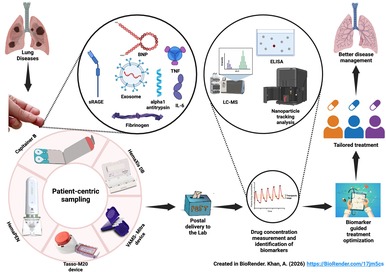

## 1 Introduction to Biomarker Use in Respiratory Medicine

Over the years, the concept of a patient‐specific therapeutic approach to disease management has become increasingly significant in respiratory medicine because of the expansion in biomarker discoveries. In biology and medicine, the acceptance of biomarker research has been swift, even if its validation and successful clinical adoption have not. Biomarkers are measurable characteristics that function as a predictor of a normal or pathogenic biological process or response to an exposure or intervention [1]. The range of respiratory markers is broad, encompassing physiological and anatomical measures such as spirometry or radiography, differential blood counts, immunological, biochemical, or nucleic acid markers. The cornerstone of respiratory disease diagnosis and monitoring remains lung function testing. Multiple assessments are required, including spirometry, fractional exhaled nitric oxide (FeNO), bronchodilator reversibility testing, and peak expiratory flow monitoring [2].

Despite their widespread use, standard diagnostic tools like spirometry and impulse oscillometry face significant clinical utility hurdles as they show high variability and technical difficulty when applied to pediatric, geriatric, or cognitively impaired populations [3]. The need for better objective criteria for diagnosis of lung disease is a long‐standing call of clinicians, as highlighted, for example, in the latest British Thoracic Society (BTS), National Institute for Health and Care Excellence (NICE), and Scottish Intercollegiate Guidelines Network (SIGN) Guidelines on asthma [2, 4, 5]. Given the inherent heterogeneity of respiratory diseases, recent research has shifted toward biomarker‐driven precision medicine to enhance both diagnostic phenotyping and long‐term treatment monitoring [6].

Several biomarkers are now integrated into clinical practice for asthma, COPD, and lung cancer ranging from cellular counts (neutrophils and eosinophils) to molecular markers like IgE, FeNO, and exhaled breath condensate (EBC) pH—to facilitate disease phenotyping and personalized respiratory therapy [7]. There is a key focus on being able to develop frameworks to tailor therapies based on biomarker‐driven individual characteristics [8]. One example is where the use of procalcitonin levels, in combination with a careful clinical evaluation, can help clinicians optimize antibiotic therapy in patients with lower respiratory tract infections and exacerbations due to chronic obstructive pulmonary disease (COPD) [9]. A further example is that while elevated total IgE levels indicate a general allergic predisposition, monitoring sIgE levels allows clinicians to tailor treatment, specifically by identifying candidates who will benefit from targeted anti‐IgE therapies (omalizumab) for chronic allergen sensitivities [10]. Fractional‐exhaled Nitric Oxide (FeNO) is an example of non‐blood‐derived biomarkers with good clinical utility in respiratory medicine. As a cost‐effective, point‐of‐care biomarker, FeNO identifies Type 2 airway inflammation and serves as a diagnostic adjunct for suspected asthma. While its lower sensitivity makes it unreliable for ruling out a diagnosis, it effectively monitors inflammation and predicts responsiveness to inhaled corticosteroids, assessing adherence to therapy and eligibility for advanced biological therapies [11].

Many of the non‐blood‐derived biomarkers have poor specificity and show variability among individuals [8]. Effective spirometry and lung function testing are highly dependent on the patient's ability to develop an appropriate technique, which is highly challenging in pediatric patients as well as in those with compromised lung function [10]. Cell counts from sputum involve complex and specialized collection, which is typically highly invasive [12]. Exhaled breath condensate and FeNO measurements, although possible in a domestic setting, are expensive and show variability [12]. There has been a resurgence over the last decade in ‘breath biopsies’, specifically volatile organic compound profiling and/or condensate from exhaled breath [13]. The appeal of the simplicity of sampling and its alignment with the practice of spirometry is obvious and it has been well reviewed over the last decade. However, despite the commercialization of high‐performing diagnostic devices with excellent instrumental and analytical validation evidence [14], as well as robust clinical studies demonstrating disease associations, clinical validation of breath biopsies has proved to be a challenge to date.

Due to the challenges in clinical validation of alternative biomarkers, there is resurgent interest in using blood‐derived protein and peptide biomarkers to identify disease phenotype specificity. While not seeking to diminish the importance of alternative biomarkers such as breath biopsies, the focus of this article is to review the developments in the field of blood‐derived biomarkers. In particular, we aim to raise awareness of a range of patient‐centric sampling (PCS) technologies that enable blood or plasma microsampling (i.e., μL volumes), and how they offer a potential solution to address the current poor translation of associational biomarkers into clinically validated biomarkers. The structure of the review is initially to assess the current approaches for blood derived biomarker measurement. Subsequently, a summary overview is provided for a range of clinically available microsampling devices before reviewing candidate biomarkers with examples of their measurement using PCS microsampling devices. The challenges and analytical considerations for the routine deployment of PCS biomarker approaches are also considered. Hence, this review aims to outline the potential that patient‐centric blood microsampling provides to develop and validate blood‐derived biomarkers for use in the diagnosis and management of respiratory diseases.

## 2 Sampling of Blood‐Based Biomarkers in Respiratory Medicine

Modern blood biomarker analysis spans the range of cell, extracellular vesicle, genomic, proteomic, and blood chemistry measurements. Cell counting in routine hematology or identification of biomarkers from plasma typically employs a large volume of sample matrix with ranges varying from 2–5 mL [15] to 10–50 mL [16]. Modern laboratory instruments and clinical blood screening require only a small volume of sample, but the automated machines to perform that analysis still employ 5–7 mL blood tubes as standard. Despite the ability of academic health science clinical researcher (typically the first adopters of biomarker advancements) to analyze 1–2 mL using analytical techniques such as ELISA and flow cytometry, routine clinical practice still remains focused on collecting standard larger‐volume tubes (2–5 mL), resulting in the routine withdrawal of significantly more blood than is technically necessary for the requested assay [15]. Traditional blood collection via venipuncture presents additional challenges, including accessibility for people living in rural and remote areas due to the need for trained personnel, the unsuitability of frequent sampling protocols in longitudinal research (due to their invasive nature), and sample collection difficulty in pediatrics and geriatrics.

While laboratory advances are enabling biomarker analysis using blood draws as low as 2–5 mL, clinically the detection of respiratory biomarkers has required the sampling of relatively large volumes of biological matrix using comparatively invasive techniques such as bronchiolar‐alveolar lavage. In recent years, novel micro‐sampling tools have been developed, which are being increasingly used for biomarker detection in biological matrices [4]. These tools can usually be used by patients themselves without recourse to specialist facilities, and as such, are leading to the emergence of patient‐centric sampling in healthcare [5]. Micro‐sampling‐driven miniaturization of sampling platforms has the potential to bypass the latter limitations and so are termed PCS solutions. PCS is an approach for obtaining high‐quality biological samples (e.g., blood, plasma, urine, etc.) from humans at any time and in any location for the determination of circulating concentrations of molecules of interest or the examination of cell types and quantities [5]. PCS, in which samples are collected at home and delivered to a centralized laboratory for analysis, gained widespread acceptance by patients and clinicians during the COVID‐19 pandemic [5]. PCS offers substantial opportunities for clinicians and clinical researchers to address the limitations of current sample collection approaches and to enhance the data richness of truly longitudinal biomarker analysis. Nevertheless, the transfer of existing sampling and assay approaches for biomarkers to microsampling techniques is not straightforward, and the nature and context of methodology transfer and its limitations will be considered in Section 6, below.

## 3 Micro‐Sampling: An Emerging Patient‐Centric Tool in Biomarker Investigations

Micro‐sampling refers to drawing small samples of biological matrices (blood, plasma, serum, urine, breast milk, saliva) from patients, usually in the range of 10 to 100 μL, in a minimally invasive way. Micro‐sampling has seen applications across diverse areas of bioanalysis, including pharmacokinetic studies, therapeutic drug monitoring, medication adherence monitoring, biomarker identification, proteomic studies, and supporting clinical trials. Microsampling enables a patient‐centric approach to monitoring, allowing for self‐sampling at home and transport via regular mail service, which significantly reduces clinic‐related costs and phlebotomy requirements. As an example, analytical agreement of validated patient‐centric microsampling with traditional venous sampling across diverse patient groups was demonstrated for tacrolimus, mycophenolic acid, and biologics such as infliximab [17]. The remote capability to analyze such high‐stakes medicines would be particularly transformative for medication adherence monitoring (MAM) and therapeutic drug monitoring (TDM). Furthermore, emerging research into biomarkers suggests that microsampling is poised to revolutionize long‐term chronic disease management and early‐stage pathological detection.

**FIGURE 1:**
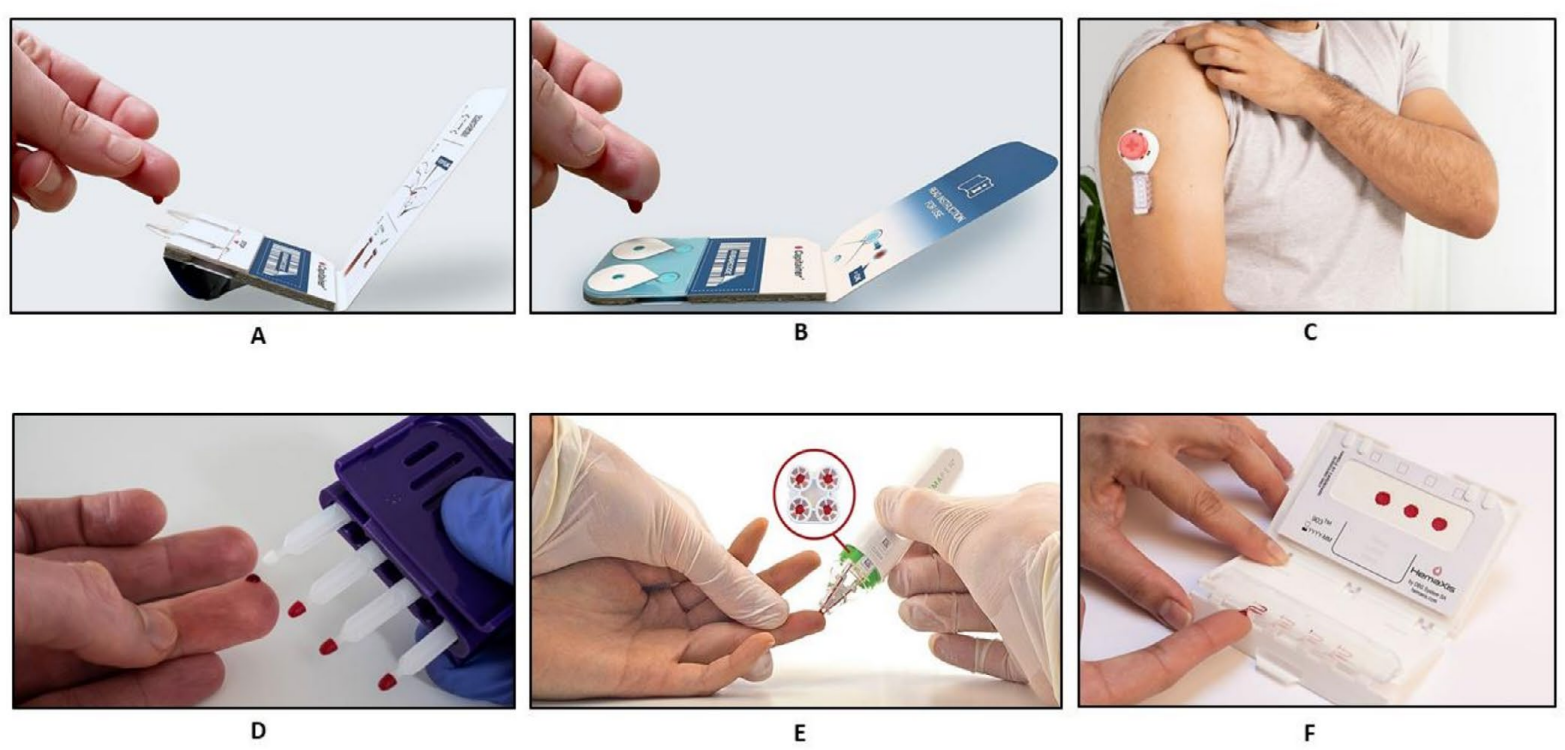
Overview of the novel micro‐sampling devices: (A) Capitainer B device (Courtesy of Capitainer, reproduced with permission from Capitainer, Sweden); (B) Capitainer B50 device (Courtesy of Capitainer, reproduced with permission from Capitainer, Sweden); (C) Tasso M 20 device (Courtesy of Tasso, reproduced with permission from Tasso); (D) Mitra device (Courtesy of Trajan Scientific and Medical, reproduced with permission from Trajan Scientific and Medical, USA); (E) HemaPEN (Courtesy of Trajan Scientific and Medical, reproduced with permission from Trajan Scientific and Medical, USA); (F) Hemaxis DB device (Courtesy of DBS system, SA, reproduced with permission from DBS system, SA, Switzerland).

Over the past 2 years, there has been a notable increase in the development of novel microsampling tools. As of 2025, a total of 50 tools were in development, with a comprehensive list accessible on the Patient‐Centric Sampling website [5]. In line with the focus of this paper, Figure 1 specifically highlights devices that have been used in the clinic in recent years. Biomarker quantification via remote microsampling could be advantageous in making clinical decisions and tailoring the medications for individual patient needs. Microsampling devices have been employed for a wide range of applications, including drug concentration measurements, doping analysis, pharmacokinetic profiling, toxicological screening, stability studies and omics‐related research [5]. The Mitra device has been the most extensively studied for biomarker investigations, followed by Capitainer cards. This could reflect the longer research history of Mitra, which was developed earlier than other microsampling devices. Notably, the use of Capitainer devices for biomarker investigations has seen a significant rise in recent years, reflecting their cost‐effective and convenient features. A summary of the published literature demonstrating the utilization of novel microsampling devices for biomarker studies, including respiratory biomarkers, analytical platforms, and the choice of matrix, is highlighted in Table S2 as supplementary data.

## 4 Blood‐Derived Biomarkers in Respiratory Diseases

A wide range of biomarkers associated with respiratory conditions, including COPD, asthma, acute lung injury, and respiratory distress syndrome, have been investigated [18]. Blood biomarker profiling has identified several potential COPD biomarkers, including lung‐derived proteins such as clara cell protein‐16 (CC‐16), surfactant protein‐D (SP‐D), and chemokine ligand‐18 (CCL‐18), markers of extracellular matrix degradation like matrix metalloproteinases (MMPs) 8 and 9, as well as systemic inflammatory biomarkers including C‐reactive protein (CRP), interleukin (IL)‐6, and IL‐8 [19]. It is achievable to “phenotype” individuals with lung diseases using these blood biomarkers and tailor therapies to the individual biomarker profile [19]. This review highlights explicitly biomarkers that are amenable to analysis using micro‐sampling techniques from blood samples. The peer‐reviewed clinical evidence supporting each biomarker, along with the analytical methods and biological matrices used for their measurement, is summarized in Table 1. The full literature used to develop the summary Table 1 is reported in Table S1 for the benefit of readers with a detailed interest.

**TABLE 1:** Peer‐reviewed clinical evidence supporting respiratory biomarkers, along with the analytical methods and biological matrices used for their measurement.

Biomarker	Disease involved	Analytical approach and sample matrix	Peer‐reviewed clinical evidence for use in clinical decision making
Eosinophils/activation markers	COPD, AECOPD	ELISA, flow cytometry, LC–MS/MS, Automated hematology analyzer Sample matrix—Whole blood, lung tissue	Extensive clinical evidence demonstrates that blood eosinophil counts are highly predictive of a patient's therapeutic response to inhaled corticosteroids (ICS) and inhaled short‐acting beta‐2 agonists Clinical decision—Therapeutic Selection Precision clinical outcome—Predicts responsiveness to Inhaled Corticosteroids (ICS) in COPD and guides biologic choice (anti‐IL5) in severe asthma
Neutrophils and their derivatives (Neutrophil elastase)	Asthma, COPD, Influenza, Bronchiectasis	ELISA, LC–MS/MS, Flow cytometry Sample matrix—Whole blood, serum, plasma, sputum	Persistent neutrophilic infiltration represents a hallmark of various chronic inflammatory lung conditions, most notably bronchiectasis, cystic fibrosis, asthma, and chronic obstructive pulmonary disease. Point‐of‐care testing for neutrophil elastase (NE) could streamline its adoption as a clinical biomarker, directly informing real‐time therapeutic strategies. A retrospective study identified the Neutrophil/Lymphocyte Ratio as a marker of severe Influenza during the 2024–2025 outbreak in France Clinical decision—Steroid responsiveness, Risk stratification Precision clinical outcome—Indicates steroid‐resistant phenotypes. In asthma, if a patient has high sputum neutrophils but low eosinophils, clinicians may decide not to escalate inhaled corticosteroids (ICS) and instead look for underlying infections or environmental triggers. Additionally, guides the incorporation of macrolides for reducing the frequency of flare‐ups in individuals suffering from chronic respiratory conditions
Immunoglobulin E (IgE)	Asthma, Cystic fibrosis, Allergic rhinitis	Fluorescent Enzyme Immunoassay (FEIA), LC–MS/MS Sample matrix—Whole blood, serum, plasma	IgE is the definitive biomarker for phenotyping allergic airway diseases, guiding the selection and dosing of targeted biologics like omalizumab. Longitudinal monitoring of IgE identifies the T2‐high inflammatory trait, enabling “steroid‐sparing” decisions that significantly reduce exacerbations and optimize long‐term clinical outcomes Precision clinical outcome—IgE levels are used to differentiate between airway diseases of allergic and non‐allergic origin. Also, IgE levels indicate whether a patient is a candidate for anti‐IgE therapy (Omalizumab/Xolair). Additionally, Omalizumab dosing is calculated using a precision medicine formula based on the patient's baseline Total IgE and body weight
Exosomes	COPD, Lung cancer, TB, ILD.	QTOF‐LC–MS/MS, flow cytometry Sample matrix‐ Serum, plasma, saliva, urine	Studies have revealed that exosomes have a cardinal role to play in the pathogenesis of COPD, including the development of emphysema, chronic bronchitis, lung cancer, tuberculosis, and interstitial lung disease. Extraction of exosomes from human plasma, urine, saliva, amniotic, and cerebrospinal fluids for global exosomal metabolome profiling has also been done Clinical decision—Liquid Biopsy Precision clinical outcome—Monitoring tumor‐derived microRNA or DNA in exosomes to track lung cancer mutations and resistance to tyrosine kinase inhibitors
Periostin	Asthma, Idiopathic Pulmonary Fibrosis (IPF)	ELISA, Electrochemiluminescence assay—Elecsys Periostin assay (Roche Diagnostics, Penzberg, Germany), LC–MS/MS Sample matrix—Serum (gold standard), BALF, sputum	Research has established periostin as a key biomarker for assessing eosinophilic inflammation, subepithelial fibrosis, and structural airway remodeling in various respiratory conditions. Furthermore, emerging evidence indicates that monomeric periostin may offer superior sensitivity for monitoring Idiopathic Pulmonary Fibrosis (IPF) than total periostin, as the latter includes multimeric variants often linked to bone metabolism rather than lung pathology. Furthermore, differential diagnosis relies on a combinatorial approach along with other markers like KL‐6 for IPF, or FeNO and IgE for Th2‐ high asthma Clinical decision—Phenotypic‐driven biologic selection Precision clinical outcome—Elevated serum periostin serves as a definitive marker for the T2‐high phenotype, identifying patients with eosinophilic inflammation and structural airway thickening. These high levels are clinically used to predict superior therapeutic responses to IL‐13 inhibitors (e.g., Lebrikizumab) and anti‐IgE biologics (e.g., omalizumab), leading to significantly improved lung function outcomes
Alpha 1 antitrypsin	COPD	Immunoassay, LC–MS/MS, capillary electrophoresis Sample matrix—Serum, plasma, stool, lung tissue	Patients with severe‐deficient genotypes account for severe AATD with clinically significant emphysema and need Alpha‐1 Antitrypsin (AAT) augmentation therapy to prevent emphysema from advancing. Oxidized AAT was a biomarker for smoke‐induced COPD and for evaluating the potential of antioxidant therapy in COPD. Elevated levels of plasma desmosine and isodesmosine correlate with decreased lung function in AATD‐induced COPD, including those with normal spirometry. Clinical decision—Disease Management Precision clinical outcome—The identification of deficiency necessitates the implementation of augmentation therapy to prevent emphysema from advancing and calls for the screening of family member
Plasma fibrinogen	COPD comorbid with cardiovascular disease	Clotting rate assays, pro‐thrombin derived, ELISA Sample matrix—Plasma	Plasma fibrinogen is associated with coronary artery diseases, vascular and non‐vascular mortality, stroke, and metabolic syndrome as well, and can identify co‐morbidities associated with COPD. Clinical decision—Risk Stratification Precision clinical outcome—Elevated levels identify COPD patients who are at increased risk for recurrent exacerbations, requiring more proactive preventive management. Additionally, elevated fibrinogen levels could be a major contributor to the development of co‐morbidities like coronary artery disease associated with COPD
sRAGE	COPD, Covid‐19	ELISA, LC–MS/MS Sample matrix—Serum, plasma, Lung tissue	sRAGE is a promising blood biomarker for COPD when combined in a panel of other blood biomarkers, including progression of emphysema and reduced lung function. The relationship to disease progression is dependent on the presence of genetic polymorphism Clinical decision—Assessment of alveolar injury Precision clinical outcome—Predicts the progression of emphysema and ARDS in high‐risk patients. Also, differentiates between hypo‐inflammatory and hyper‐inflammatory sub phenotypes
C‐RP	Systemic inflammation, COPD, CVD, Lung cancer	ELISA, LC–MS/MS, Nephelometry Sample matrix—Serum, plasma, sputum, lung tissue, exhaled breath condensate	Elevated C‐RP levels are linked to a higher risk of mortality and severe exacerbations in COPD patients, as well as identifying underlying inflammatory activity in smokers without the disease. Studies have shown that antibiotic prescribing guided by C‐RP levels during COPD exacerbations led to a reduction in both patient‐reported antibiotic use and clinician‐issued prescriptions, without any indication of adverse effects Clinical decision—Antimicrobial Stewardship Precision clinical outcome—Utilized in primary care to effectively minimize unwarranted antibiotic prescriptions for acute exacerbations of COPD
IL‐6 and IL‐8	Lung cancer, Covid‐19, ILD	Electrochemiluminescence immunoassay (ECLIA), flow cytometry Sample matrix—Serum, plasma, sputum, nasal lavage	High level of serum IL‐6 in ILD could predict acute exacerbation and poor prognosis in patients with ILD, and predict COVID‐19 severity, although IL‐8 was a more sensitive biomarker for COVID‐19. Serum IL‐6 and IL‐8 levels were potential diagnostic biomarkers for lung cancer Clinical decision—Immunomodulation, diagnosis and severity tracking Precision clinical outcome—High levels of IL‐6 identify patients with COVID‐19 or ARDS who will benefit from IL‐6 receptor antagonists like Tocilizumab. IL‐8 acts as a marker of neutrophilic inflammation and mucus hypersecretion is used to monitor cystic fibrosis and bronchiectasis
Serum surfactant proteins‐ A and D	COPD, COVID‐19, COVID‐19‐like pneumonia, RDS, IPF, cystic fibrosis	ELISA, chemiluminescent enzyme immunoassay (CLEIA) Sample matrix—Serum, plasma, lung tissue	Association between elevated serum SP‐D levels and increased severity of COVID‐19. Lower serum levels of SP‐D and Krebs von den Lungen (KL‐6) could distinguish COVID‐19 pneumonia from that of COVID‐19 pneumonia‐like disease Clinical decision—Barrier integrity Precision clinical outcome—Elevated serum levels indicate damage to the alveolar‐capillary barrier, specifically in Idiopathic pulmonary fibrosis (IPF)
Brain Natriuretic peptide (BNP) and NT‐pro BNP	COPD comorbid with CVD	LC–MS/MS, Immunoassays (e.g., TriageBNP, ADVIA Centaur BNP, AxSYM BNP assay) Sample matrix—Serum, plasma	Elevation of serum BNP and NT proBNP levels in COPD patients confirmed by metaanalysis, and in pulmonary hypertension in COPD patients. Increase in the BNP/NT proBNP occurs during COPD exacerbation offering differential diagnostic biomarker for acute dyspnea of cardiac or pulmonary origin and decide optimum therapy. BNP testing improved the detection of chronic heart failure in COPD patients by 20%, including in patients with no history of heart failure Clinical decision—Differential Diagnosis Precision clinical outcome—Quickly distinguishes acute heart failure from pulmonary origins (Asthma/COPD) in patients with acute dyspnoea. Serum levels of both BNP and NT‐proBNP can also be utilized as a predictive biomarker in pulmonary hypertension
CCL‐18	COPD	ELISA Sample matrix—Serum, plasma, lung tissue	Serum concentrations of CCL‐18 were significantly higher in COPD patients than in healthy individuals, and that there was a correlation between higher levels of CCL‐18 in serum and the number of COPD exacerbations. Serum CCL‐18 level was predictive for the application of inhaled corticosteroids in hospitalized COPD patients Clinical decision—Prognostication Precision clinical outcome—Elevated serum level is among the most dependable indicators of mortality and lung function deterioration in IPF
TNF‐alpha	COPD, asthma, ARDS, sarcoidosis, and ILD	Immunoassays, Flow cytometry, ELISA Sample matrix—Serum, plasma, sputum, exhaled breath condensate	A meta‐analysis study found that serum TNFα levels were significantly higher in the COPD group than in the control group, and both TNF‐α and interleukin (IL‐1β) were elevated in patients with COPD, highlighting their role as biomarkers for COPD and the severity of airflow limitation Clinical decision—Disease phenotyping Precision clinical outcome—Identifies “High‐Inflammatory” phenotypes at risk for rapid FEV1 decline and frequent hospitalizations
Pro calcitonin (PCT)	Community‐acquired pneumonia (CAP) and other bacterial respiratory infections	TRACE (Time‐resolved amplified cryptate emission technology assay Kryptor PCT; Brahms AG) Sample matrix—Serum, plasma	PCT has been widely used to guide antibiotic treatment and PCT assays has been approved by the USA Food and Drug Administration to guide antibiotic treatment in respiratory tract infections. A meta‐analysis study showed PCT‐based antibiotic treatment lowers antibiotic exposure and side effects, and improved survival, while PCT has shown potential to distinguish between bacterial or viral respiratory infections Clinical decision—Infection Control Precision clinical outcome—Guiding the initiation and more importantly the duration of antibiotic therapy in lower respiratory tract infections

### 4.1 Eosinophils

In COPD and asthma, eosinophils function as key effectors of airway pathology by degranulating and releasing cytotoxic mediators, which consist of chemokines and proteins. These are instrumental in driving the chronic inflammation, structural airway remodeling, and bronchial hyperresponsiveness that characterize the clinical phenotype of the disease [20]. Peripheral eosinophil counts have established themselves as a premier biomarker in the clinical management of COPD and, in particular, asthma, primarily due to their accessibility and their role in reflecting Type 2‐high airway inflammation [6] and to guide treatment to inhaled corticosteroids and biologics [7]. Although the correlation between systemic levels and localized tissue infiltration is not absolute, blood eosinophil concentrations serve as a reliable surrogate for eosinophilic activity within the lungs. For example, GOLD guidelines suggest that COPD patients with eosinophil count ≥ 300 cells/μL of blood are most likely to benefit from inhaled corticosteroid treatment [10].

While blood eosinophil counts are vital biomarkers, their clinical reliability is often compromised by diurnal fluctuations (peaking at midnight and troughing at midday) as well as influences from infections, medications, and comorbidities [21]. Research indicates that higher baseline variability in these counts is directly linked to an increased risk of COPD exacerbations, necessitating diagnostic strategies that account for these temporal shifts [20]. To optimize clinical outcomes in eosinophilic COPD, diagnostic frameworks must account for the inherent variability of eosinophil levels by prioritizing non‐invasive, longitudinal monitoring [20]. This emphasizes the need for patient‐centric microsampling technologies that could effectively mitigate this issue by enabling high‐frequency, non‐invasive sampling to capture a more accurate inflammatory profile and optimize personalized treatment protocols.

Eosinophil counts in COPD are measured using a hierarchy of diagnostic techniques ranging from systemic surrogates to direct tissue sampling. Fractional exhaled nitric oxide (FeNo) and exhaled breath condensate (EBC) are non‐invasive methods and serve as indirect physiological markers of airway eosinophilia [22]. Measuring eosinophil activity from microsampling is currently achievable. The most validated device for measuring absolute blood eosinophil counts (BEC) from a finger‐prick is the HemoCue WBC DIFF System, which uses only 10 μL of capillary blood with a very close correlation (*r* = 0.90 to 0.98) between the HemoCue finger‐prick device and standard laboratory analyzers (like the Abbott Architect) in patients with asthma and COPD [15]. Measuring the absolute eosinophil count from dried blood spot devices like the Capitainer or VAMS presents significant challenges given the dried nature of the matrix; however, measuring cell markers like eosinophil cationic protein (ECP) serves as a viable alternative, acting as a stable biochemical surrogate for eosinophil activity even in dried micro‐volume samples [23].

### 4.1 Alpha 1 Antitrypsin

Alpha‐1 antitrypsin (AAT) is a plasma glycoprotein and a serine protease inhibitor [18]. About 1%–2% of the COPD population is reported to have Alpha‐1 Antitrypsin deficiency (AATD) [24]. This condition causes a loss of systemic balance between protease and antiprotease activity and the lack of a fundamental anti‐inflammatory acute‐phase factor [24]. In the pathogenesis of COPD, the protective role of AAT lies in the inhibition of two protease enzymes: neutrophil elastase and proteinase‐3. Neutrophil elastase has positive effects when expressed at normal levels. However, when overexpressed, it can cause loss of epithelial cell integrity and their shedding via excessive degradation of extracellular matrix proteins such as elastin, collagen, and fibronectin, as well as cell‐associated proteins such as E‐cadherin [25]. This results in disruption of the epithelial barrier and degradation of lung parenchyma. Disinhibition of these processes causes pathophysiological changes of emphysema and persistent inflammation [25]. Hepatocytes and bronchial epithelial cells primarily produce AAT to protect tissues and modulate inflammation via the LRP1 receptor.

Various studies performed on plasma and urine samples of humans and animals have linked elevated levels of elastin degradation products desmosine and isodesmosine (the latter being the major degradation product) with decreased lung function in AATD induced COPD [26]. Typical sample volumes reported for AATD pathway monitoring are 50 mL of blood from each subject. This is an area where micro‐sampling could play a transformational role. These biomarkers have been rigorously analyzed using a range of complementary platforms including immunoassays for high‐throughput screening, LC–MS/MS for precise quantification, and capillary electrophoresis for complex metabolite separation [27]. Microsampling‐based dried blood spot (DBS) methods are available in the literature for AATD screening with a correlation coefficient of 0.8674 (*p* < 0.0001) between alpha1‐AT concentrations in DBS versus serum samples [28].

### 4.2 Plasma Fibrinogen

Fibrinogen, a glycoprotein synthesized in hepatocytes, acts as a coagulation factor when released into the systemic circulation, and its inflammatory role as an acute‐phase reactant makes it a suitable biomarker in COPD [19]. Individuals who report plasma fibrinogen levels of ≥ 350 mg/dL are at a higher risk of COPD exacerbation and mortality [29]. Plasma fibrinogen was given approval in 2015 by the United States Food and Drug Administration (USA FDA) for its role as a predictive biomarker for clinical trial enrichment in submissions for investigational new drug applications, new drug applications, and biologics license applications. The successful validation of plasma fibrinogen and its acceptance by USA FDA as a predictive biomarker for drug development in COPD was a significant step toward facilitating clinical trials for the development of novel drugs [30]. Plasma fibrinogen could be a major contributor to the development of co‐morbidities associated with COPD. This is due to the fact that fibrinogen is found to be associated with coronary artery diseases, vascular and non‐vascular mortality, stroke, and metabolic syndrome as well [31]. A microsampling‐based assay called the dry‐hematology method (DRIHEMATO) is available to measure fibrinogen levels in plasma and whole blood, utilizing 20 μL of plasma or whole blood [32]. Research indicates that fibrinogen concentrations measured via the dry hematology method were significantly lower (87.9% ± 3.1%) compared to those obtained using the gold‐standard Clauss method. This discrepancy suggests that clinicians should exercise caution, as the presence of heparin may artificially suppress fibrinogen readings when using dry reagent microsampling techniques [33].

### 4.3 Biomarkers From the Inflammatory Cascade

#### 4.3.1 C‐ Reactive Protein (C‐RP)

C‐RP is an acute‐phase protein that is mostly produced by hepatocytes in response to bacterial infections, inflammatory events, and tissue damage. Serum C‐RP levels are typically low (usually < 1 mg/dL) in healthy individuals. However, in response to stressors such as bacterial infections or tissue injury, hepatocytes rapidly synthesize and release large quantities of C‐RP into the bloodstream, with levels rising about 1000‐fold above the normal [34]. Research findings reveal that antibiotic prescribing guided by C‐RP levels for COPD exacerbations led to a reduction in both patient‐reported antibiotic use and clinician‐issued prescriptions, without any indication of adverse effects [35]. These findings highlight the potential of C‐RP in optimizing antibiotic therapy in COPD management, paving the way for personalized treatment plans. Methods for assessing C‐RP levels include LC–MS/MS‐based assay, enzyme‐linked immunosorbent assay (ELISA), lateral flow immunoassay (LFIA), and immunoturbidimetry (ITM) based assay [36]. Microsampling‐based methods are available in the literature, providing evidence that C‐RP levels from a finger‐prick microsample are nearly identical to those from a traditional venous draw [37]. For example, one study showed a correlation coefficient of 0.986 between finger‐prick microsampling and traditional venous draws with 100% sensitivity to classify cardiovascular disease risk [38]. Another study has demonstrated that C‐RP measurements across serum, plasma, and dried blood spots exhibit exceptional Pearson correlations (ranging from 0.974 to 0.995) [39].

#### 4.3.2 Interleukin‐6 (IL‐6) and Interleukin‐8 (IL‐8)

Interleukins (IL) are cytokines that have been shown to be expressed by a variety of different bodily cells, required for immune cell activation, differentiation, proliferation, maturation, migration, and adhesion, in addition to being anti‐inflammatory and pro‐inflammatory. Interleukins, particularly IL‐6 and IL‐8, play a critical role as central drivers of pathogenesis across a spectrum of respiratory conditions, including COPD, asthma, ARDS, lung cancer, and interstitial lung diseases. Studies have also demonstrated the role of interleukins as a potential biomarker for diagnosing and managing lung‐related disorders. For example, in COPD, serum IL‐6 and IL‐8 levels have been reported to be significantly higher compared to healthy individuals [40]. A retrospective study conducted from the year 2016 to 2019 of 83 patients with interstitial lung disease (ILD) confirmed that a high level of serum IL‐6 in ILD could be a useful biomarker to predict acute exacerbation and poor prognosis in patients with ILD [41]. Another retrospective study conducted on 133 patients with lung cancer highlighted the role of serum IL‐6 and IL‐8 levels as potential diagnostic biomarkers for lung cancer [42]. A study in Covid‐19 patients suggested that serum IL‐6 and IL‐8 could be potential biomarkers for predicting Covid‐19 severity and prognosis [43]. A microsampling‐based multiplex assay based on VAMS for the determination of 31 inflammatory markers, including IL‐6 and IL‐8, is available in the literature [44]. Another study demonstrates an LC–MS‐based assay for measuring low abundance proteome (including interleukins) from a 10 μL VAMS tip [45].

#### 4.3.3 TNF‐α

Tumor necrosis factor alpha (TNFα)‐ the most widely researched cytokine within the TNF superfamily is released by macrophages in response to lipopolysaccharide stimulation and promotes tumor necrosis when administered to tumor‐bearing mice. TNF‐α is a type of glycoprotein that contributes to the production of T‐lymphocytes, neutrophils, mast cells, fibroblasts, and endothelial cells. Elevated levels of TNFα have been associated with various respiratory diseases, including asthma, COPD, acute respiratory distress syndrome (ARDS), sarcoidosis, and interstitial pulmonary fibrosis (IPF) [46]. The role of TNFα in the pathogenesis of lung diseases is by promoting the accumulation of inflammatory cells, driving the production of inflammatory mediators, and inducing oxidative and nitrosative stress, airway hyperresponsiveness, and tissue remodeling [46]. Results from a meta‐analysis study revealed that serum TNF‐α levels were significantly higher in the COPD group than in the control group (SMD: 1.24, 95% CI: 0.78–1.71, *p* < 0.00001) [47]. A prospective case–control study concluded that levels of TNF‐α, along with interleukin (IL‐1β), were elevated in patients with COPD, highlighting their role as biomarkers for COPD and severity of airflow limitation [48]. TNFα is mainly analyzed by immunoassays and flow cytometry [49]. A microsampling based multiplex assay for the determination of 31 inflammatory markers including TNF‐α is available in the literature [44].

### 4.4 Brain Natriuretic Peptide (BNP) and N‐Terminal proBNP

Natriuretic peptides (NPs) have emerged as key candidates for the development of diagnostic tools and therapeutic agents in cardiovascular disease, playing a crucial role in maintaining cardiovascular and cardiorenal homeostasis. NPs consist of three primary peptides—A type, also called Atrial Natriuretic Peptide (ANP), B type, also called Brain Natriuretic Peptide (BNP), and C type (CNP) [50]. The biological effects of natriuretic peptides occur in terms of vasodilation, natriuresis, and diuresis in addition to downregulating the renin‐angiotensin‐aldosterone system [51]. Among the family of natriuretic peptides, BNP and NT proBNP are clinically relevant to respiratory diseases [51]. BNP is secreted in its precursor form called pro‐BNP, which separates into the biologically active form BNP and an inactive component called N‐terminal proBNP (NT proBNP).

A meta‐analysis study on the relevant published literature from 1990 to 2021 concluded that serum BNP and NT proBNP levels are elevated in COPD patients [50]. The study also confirmed that there is a further increase in the BNP/NT proBNP during COPD exacerbation, highlighting the potential of these peptides as a diagnostic biomarker for ruling out heart failure and for differentiating acute dyspnoea of cardiac or pulmonary origin [50]. One study documented that BNP and NT‐ proBNP are sensitive biomarkers and by implementing BNP testing, the detection of chronic heart failure in COPD patients could be improved by 20% [52]. The study also revealed that both BNP and NT‐proBNP values may be increased in COPD patients even without a history of heart failure. The study further showed that BNP itself has broncho‐relaxant and bronchoprotective properties in human airways and could be a possible COPD treatment [52]. The fact that serum concentrations of NT‐proBNP are elevated in clinical conditions affecting both the left and right ventricles of the heart, and that management of cardiovascular comorbidities in COPD is an important guideline, measuring the serum or plasma levels of BNP and/or NT‐proBNP may help guide the optimum treatment in the diagnosis, prevention, and management of cardiopulmonary diseases [53].

BNP and NT‐proBNP levels are measured using immunoassays, each employing antibodies that target different epitopes on the antigen molecules. For example, TriageBNP‐ a rapid immunofluorescent assay by Biosite Diagnostics, provides point‐of‐care results in 15 min. Additionally, the ADVIA Centaur BNP assay by Bayer Healthcare and the AxSYM BNP assay by Abbott Diagnostics are primarily laboratory‐based BNP assays [54]. Furthermore, some LC–MS/MS based assays for measuring BNP and NT proBNP are also available in the literature [55]. BNP is highly susceptible to degradation upon storage (see Section 5.3) and hence sample stability is a key pre‐analytical concern for clinical validation of a BNP or NT‐proBNP bioanalytical strategy. A microsampling‐based method utilizing the LumiraDx NT‐proBNP device (using 20 μL of capillary blood) against the laboratory gold standard (Roche Cobas) showed an exceptional Deming correlation of *r* = 0.982, suggesting it a viable alternative to venous draws in emergency departments and primary care [56]. Another comprehensive review identifies NT‐proBNP as one of the few cardiovascular biomarkers (alongside ApoB and Creatinine) that demonstrated strong laboratory validation for the dried blood spots [57]. NT‐proBNP is considered a more stable biomarker and is easier to work with compared to BNP. This has been explained in more detail under Section 6.3.

### 4.5 sRAGE

Among the novel COPD biomarkers that have been investigated in extensive longitudinal studies like ECLIPSE (Evaluation of COPD Longitudinally to Identify Predictive Surrogate Endpoints), SUMMIT (Study to Understand Mortality and MorbidITy), SPIROMICS (Sub Populations and Intermediate Outcomes In COPD Study), and COPDgene (Genetic Epidemiology of COPD), the soluble receptor for advanced glycation end‐products (sRAGE) has come out to be among the most promising blood biomarkers [58]. Receptor for advanced glycation end‐products (RAGE), a pro‐inflammatory pattern recognition cell surface receptor belonging to the immunoglobulin superfamily, plays a significant role in the innate immune response and serves as a facilitator of pro‐inflammatory processes [59]. This RAGE has an extracellular domain called sRAGE that is produced by either alternative splicing of the *AGER* gene or proteolytic cleavage of the receptor by metalloproteinase enzymes like MMP9 and ADAM10. While the membrane‐bound receptor promotes inflammation, its soluble form (sRAGE) exhibits anti‐inflammatory characteristics as it exists as a sink receptor and also prevents the homodimerization of RAGE, which is required for downstream signaling [60].

To date, sRAGE is still the most promising blood biomarker for COPD because it has been proposed in the literature that its discriminating power can be increased using a panel of other blood biomarkers [60]. However, its relationship with disease progression is erratic in the cohorts examined, as sRAGE measurements are affected by genetic polymorphism. This has been confirmed, for example, in studies, which showed that the circulating level of sRAGE is affected by the missense single‐nucleotide polymorphism (SNP) “rs2070600” on the *AGER* locus (the gene that codes for RAGE). This genetic polymorphism is present in approximately 5%–7% of the population [60]. Meta‐analyses of large longitudinal cohorts have established that systemic sRAGE levels are significantly reduced in COPD patients, serving as a robust biomarker that correlates with both the severity and the progression of emphysema [61]. sRAGE isoforms are reported to be stable enough and have been quantified in stored frozen serum or plasma samples via the antibody‐based ELISA assays [59]. An LC–MS/MS based microsampling assay for the quantification of sRAGE is available in the literature utilizing 50 μL of serum [62].

### 4.6 Serum Surfactant Proteins A and D

Surfactant proteins make up 8 to 10% of the pulmonary surfactant‐ a lipid‐protein mixture that lines and stabilizes the alveoli to facilitate gas exchange during breathing, while also serving as a primary defense against pathogens in the deep lungs. Based on their molecular weights and varied structure, they are classified into four groups namely A, B, C, and D. Among these, surfactant protein A (SP‐A) and surfactant protein D (SP‐D) exert antibacterial function and are involved in the elimination of apoptotic cells, participation in the process of immune function, inflammatory response, and lipid metabolism at the same time [63]. The involvement of SP‐A in the pathophysiological processes of respiratory diseases like COPD, respiratory distress syndrome (RDS), idiopathic pulmonary fibrosis (IPF), cystic fibrosis, mycoplasma pneumonia, and lung inflammation has been well‐documented [64].

Regarding SP‐D, the fact that it is primarily synthesized within the respiratory tract, its potential as a biomarker in people with community‐acquired pneumonia, interstitial fibrosis, drug‐induced lung disease, and allergic bronchopulmonary aspergillosis in cystic fibrosis has been widely addressed [65]. Meanwhile, in human research, both SP‐A and SP‐D have been linked to smoking and genetic variation in the pathogenesis of COPD [65]. Results from a retrospective cohort study in 54 patients with COVID‐19 pneumonia and 65 patients with COVID‐19 pneumonia‐like diseases showed that lower serum levels of SP‐D and Krebs von den Lungen (KL‐6) were associated with COVID‐19 pneumonia, showcasing the potential of SP‐D as a biomarker for distinguishing COVID‐19 pneumonia from that of the COVID‐19 pneumonia‐like disease [66]. Assays for the measurement of surfactant proteins are based on ELISA and LC–MS [67]. Surfactant proteins are highly suitable for microsampling due to their high circulating levels, even though no specific assay currently exists for this platform. The Tasso‐SST could be the preferred device for these markers as it provides liquid serum, avoiding the “trapping” or extraction issues that can occur with dried blood spots (DBS) for large, complex proteins.

### 4.7 Serum Chemokine Ligand‐18 (CCL‐18)

Chemokine Ligand‐18 (CCL‐18), also known as pulmonary and activation‐regulated chemokine (PARC)‐ a polypeptide consisting of 69 amino acids and having a molecular weight of 7.85 KDa, is highly expressed in the lungs, placenta, lymph nodes, monocytes, dendritic cells, and bone marrow [68]. The increased serum CCL‐18 levels have been found to be involved in chronic inflammation of the airways, pulmonary interstitial fibrosis, and allergic pneumonia [68]. A study performed on sixty COPD patients and 20 participants with a smoking history, but normal spirometry, concluded that serum concentrations of CCL‐18 were significantly higher in COPD patients than in healthy individuals and that there was a correlation between higher levels of CCL‐18 in serum and the number of COPD exacerbations [69]. This was further confirmed by another study involving 98 COPD patients and 60 healthy volunteers, concluding that serum levels of CCL‐18 were significantly higher in COPD patients compared to healthy individuals and that serum CCL‐18 level was predictive for the application of inhaled corticosteroids in hospitalized COPD patients [70]. Both of these studies used ELISA methods for measuring the CCL‐18 levels. The former has reported a requirement for 10 mL of blood and the latter 5 mL of blood from each of the participants for performing the assay. Although microsampling‐based assays for CCL‐18 are not yet widely established, CCL‐18 is uniquely suited for microsampling due to its high physiological concentration, averaging 80–105 ng/mL in clinical cohorts, and its robust stability in dried formats [69, 70].

### 4.8 Procalcitonin (PCT)

Procalcitonin, a prohormone of the calcium‐regulating hormone calcitonin, is primarily synthesized by neuroendocrine C‐cells in the thyroid gland under non‐infectious conditions. In healthy individuals, circulating procalcitonin levels are typically low (< 0.05 ng/mL). However, during bacterial infections, there is a marked and selective elevation of procalcitonin concentrations, particularly in the parenchymal tissues. Additionally, serum PCT levels have been found to have high specificity as a biomarker for distinguishing between bacterial and non‐bacterial inflammations [71]. In 2017, The US Food and Drug Administration approved PCT assays for use in managing sepsis and respiratory tract infections by guiding the antibiotic treatment [72]. A meta‐analysis study proved that procalcitonin‐guided antibiotic treatment in patients with acute respiratory infections lowers antibiotic exposure and side effects and improves survival [73] while another study concluded that procalcitonin‐driven therapy in patients with lower respiratory infections did not lead to a reduction in antibiotic usage [74]. Based on these observations, it is not clear whether or not procalcitonin‐guided therapy results in decreased antibiotic usage in lower respiratory tract infections. However, this does not mask the potential of PCT as a biomarker for distinguishing between the respiratory infections of bacterial and those of viral origin.

A multiplex microsampling method for measuring a panel of 17 proteins, including PCT from the serum samples of COVID‐19 patients and healthy individuals, is available in the literature. The method highlights the utilization of the Tasso‐SST microsampling device for at‐home testing [75]. However, to date, there has been no clinical validation of a microsampling approach to PCT quantification that supports home sampling for clinical decision making, in particular due to the narrow concentration thresholds for different diseases, and also due to its susceptibility to degradation [76]. Findings from one study showed that PCT levels in EDTA blood suffered a 6%–12% loss of concentration between 12 and 24 h in a laboratory setting [77]. Since PCT clinical decisions happen at ultra‐low levels (0.1 ng/mL), even a 10% loss will be significant. Therefore, home‐sampled PCT monitoring cannot yet be used to safely “rule out” sepsis without further longitudinal stability studies.

### 4.9 Periostin

Periostin is a matricellular protein detectable in serum and serves as a potent indicator of airway eosinophilia in severe asthma [78]. Notably, the gene coding for periostin is one of the most highly up‐regulated genes in asthmatic patients, with its expression in airway epithelial cells directly modulated by the key Type 2 (T2) cytokines‐ IL‐4 and IL‐13 [78]. Evidence suggests that periostin functions as more than a mere surrogate biomarker for Type 2 immunity because it identifies a unique clinical asthma phenotype characterized by late‐onset disease, aspirin intolerance, nasal comorbidities, elevated FeNO, and pronounced eosinophilia [79]. These traits likely stem from the fact that periostin has an active role in structural tissue remodeling rather than just acute inflammation [79]. Consequently, periostin serves a dual role in precision diagnosis by acting as a systemic indicator of Type 2‐driven inflammation while simultaneously signaling airway remodeling that often results in corticosteroid hyperresponsiveness [79].

A cross‐sectional study on 76 adults, with 25 healthy controls, 25 moderate, and 26 severe asthma patients, concluded that Serum periostin functions as a robust diagnostic biomarker for asthma, enabling the identification of distinct, clinically relevant phenotypes through multidimensional clustering [80]. By integrating periostin levels with inflammatory burden and lung function data, clinicians can better characterize patient subsets, directly facilitating more precise and personalized asthma management strategies [80]. Periostin also plays a key role in the pathogenesis of idiopathic pulmonary fibrosis (IPF) by acting on fibroblasts along with inflammatory cytokines such as TNF‐α or IL‐1α, activating NF‐κB, followed by production of various inflammatory cytokines and chemokines. All of which leads to the generation of fibrosis in the lungs [81]. A study done by Shoichiro Ohta et al. reported the monomeric‐to‐total periostin ratio to be significantly higher in IPF than in other periostin‐elevated conditions [81]. This suggests that quantifying the monomeric isoform could serve as a superior diagnostic tool for IPF and a more precise predictor of its short‐term clinical progression.

A key finding in longitudinal monitoring is that periostin levels show a slight diurnal rhythm, with peak levels in the morning. However, the magnitude of this change is usually too small to shift a patient's clinical classification (e.g., from “T2‐high” to “T2‐low”) [82]. For the measurement of serum periostin levels, the gold standard is the Elecsys Periostin assay (Roche Diagnostics, Penzberg, Germany). Other methods include sandwich ELISA [81]. A top‐down LC–MS/MS‐based proteomic assay is also reported in the literature for the specific identification of human periostin isoforms [83].

## 5 Gaps in the Clinical Validation of Blood‐Derived Respiratory Biomarkers

While clinicians rely on ‘validated’ biomarkers for respiratory conditions, a notable disparity exists between the presumption and reality of clinical validation. Despite the academic literature being rich with analytical method validation and association studies in appropriate patient groups for a host of biomarkers [84], as few as 100 out of 150,000 reported biomarkers reached suitability and acceptance for routine use in the clinic [85]. An analysis of the USA Food and Drug Administration Biomarker Database reveals as few as 14 entries in the broad disease therapy area of pulmonology, not all of which have been accepted as drug development tools (US‐FDA CDER & CBER Drug Development Tool Qualification Project Search‐ https://force‐dsc.my.site.com/ddt/s/). Association studies are generally performed in isolated clinical study sites due to the local clinical interest and availability of analytical technologies. The challenge in biomarker validation rests in the fact that the literature abounds with biological analyses performed on stored frozen samples, while regulatory validation steps will require sample stability to be demonstrated between sampling and storage, and analytical technique robustness between labs and measurement techniques that supports clinical statistical validation [86]. Considering the patient experience, biomarker studies provide data points with poor time resolution (i.e., follow‐up clinic visits), obtained at variable times of day when they attend a clinic, often due to a disease‐relevant event.

Most validation studies in respiratory medicine are conducted within the rigid constraints of a clinical setting, typically during office hours, creating a circadian blind spot where pathology is often defined by nocturnal or early‐morning timing [87]. For example, eosinophil counts in sputum and serum IgE show distinct peaks at 4:00 AM—a time when patients are rarely sampled in a clinical setting [87]. In one study, the authors explicitly compared morning versus afternoon clinic results and found that 37.4% of patients had positive sputum eosinophil counts (≥ 3%) in the morning, while only 21.6% in the afternoon. This proves that sampling time directly influences clinical management and can lead to the underestimation of inflammatory burden. Hence, investigating the circadian variability is crucial for the validation of respiratory biomarkers, and the diurnal fluctuations of any prospective biomarker must be measured and regulated in both research and clinical settings [88]. This is where patient‐centric microsampling has proven promising by removing the need for a clinic visit and allowing for the capture of real‐world biological oscillations. For example, a seminal study from the Mike Snyder group (from Stanford) explicitly demonstrated dense, high‐frequency microsampling (hourly/daily) for multi‐omics profiling and proved that collecting micro volumes (< 50 μL) remotely reveals large‐scale molecular fluctuations that are completely missed in traditional clinical snapshots [16].

## 6 Decision‐Grade Parameters To Be Validated for Biomarker Monitoring via Microsampling

The advancement in novel microsampling tools has revolutionized bioanalysis by enabling minimally invasive, low‐volume blood collection [89]. However, as recommended by the USA FDA Guidance (2025), for the successful implementation in routine clinical care, researchers must move beyond proof‐of‐concept, and the validation rigor must scale significantly, covering stability, reproducibility, and preanalytical handling in ways that exploratory studies do not [1]. Some of the important decision‐grade validation parameters that need to be investigated while developing biomarker assays on microsampling devices are hematocrit bias, choice of matrix, at‐home user technique, analyte stability, shipping conditions, and suitability for the assay.

### 6.1 Hematocrit Bias

VAMS and Mitra devices can suffer from hematocrit (HCT) bias, where variations in blood viscosity resulting from variation in red blood cell content can affect sample absorption, leading to inconsistencies in analyte concentrations [90]. Capitainer attempts to address this issue by metering a fixed blood volume through the channels. This has been confirmed in an analysis of 133 blood samples from hospital patients at different HCT levels for the quantification of caffeine and paraxanthine [91]. A similar study utilizing the Tasso M20 device for the quantification of nirmatrelvir from dried human blood showed that HCT levels had no impact on the analyte recovery [92]. In dried blood spot microsampling, HCT variation among patients may induce measurement errors due to the inability to separate plasma and cytoplasm of blood cells, especially if the biomarker is present in the plasma fraction of blood, like with C‐RP [93]. Researchers tested blood samples with three different C‐RP levels (3.6, 8.6, and 50 mg/L) across a wide range of HCT (16%–55%). They found that as the percentage of red blood cells increased, the measured CRP level dropped. For example, at the highest C‐RP level, every 1% increase in HCT caused the CRP reading to decrease by 0.19 mg/L [38].

### 6.2 Capillary Versus Venous Blood and the Use of Conversion Factors

Capillary blood can be a useful alternative to venous blood for biomarker analysis [94]. However, a direct 1:1 correlation may not always be the case for biomarkers (e.g., the protein S100B) [95]. In one study on the measurement of C‐RP levels, the concentrations in serum were found to be, on average, 1.6 times higher (SD 0.37) than those measured in dried blood spots [39]. In another study evaluating 17 different proteins, researchers found that the majority of biomarkers, including C‐RP, Ferritin, IL‐6, and PCT, showed strong correlation and minimal bias between Tasso‐SST capillary microsamples and traditional venous serum. In contrast, D‐dimer, IL‐1B and IL‐1Ra concentrations did not correlate well [75]. These findings highlight that clinical decision‐making must be based on a quantified bias adjustment. For protein biomarkers, a Deming or Passing‐Bablok regression is required to develop conversion factors. For example, one study examining the comparability of venous and capillary blood samples for 34 chemistry analytes revealed that Deming regression and mean relative differences demonstrated excellent comparability between venous and capillary samples for most measured analytes [94].

### 6.3 Biomarker Stability

Certain biomarkers, including unstable proteins and lipids, may degrade more rapidly in dried samples compared to liquid plasma or serum. This has been confirmed by a 2024 study assessing protein stability in dried blood spots (DBS) stored at room temperature for up to 6 months [96]. The study revealed that approximately 4% of detected proteins exhibited significant changes during the drying process, predominantly affecting cytoplasmic proteins. Additionally, 19 proteins displayed temporal instability during storage, with cytoskeleton‐related proteins declining and proteins involved in cytoplasmic transport increasing over time. The study concluded that protein instability in DBS samples is primarily associated with hydrophilic proteins and is exacerbated by prolonged storage durations [96]. A quintessential example of molecular instability in the context of remote diagnostics for respiratory biomarkers is the contrast between BNP and NT‐proBNP. BNP is highly susceptible to proteolytic degradation, exhibiting a 10%–15% decline within 2 h at room temperature [97]. Furthermore, BNP recovery has been reported to drop by 70% in frozen EDTA plasma after just 1 day of storage [98]. NT‐proBNP, on the other hand, is significantly more stable at room temperature for up to 72 h and can be stored at −20 degrees for months without significant loss of immunoreactivity. These differences make NT‐proBNP a preferred biomarker for unmonitored remote sampling [98].

### 6.4 Pre‐Analytical Caveats

Pre‐analytical errors during at‐home sampling as well as shipping represent a significant risk to data integrity. For example, cytokine levels for IL‐6 can be artificially inflated by the release of interstitial fluid if the finger is ‘milked’ or squeezed excessively during collection [99]. This necessitates the use of standardized SOPs, such as hand‐warming, to promote spontaneous blood flow. One study identified outdoor courier lockboxes in summer to be a significant source of preanalytical error [100]. The study concluded that blood samples stored in outdoor courier lockboxes during summer experienced significant changes in analytes, such as aspartate aminotransferase, glucose, lactate dehydrogenase, and potassium, due to high temperatures [100]. These findings highlight the importance of evaluating analyte stability at extreme temperatures depending on the region.

A targeted study evaluating the stability of 34 metabolic markers in DBS samples revealed that high humidity and high temperatures were the primary drivers of biomarker degradation in dried blood spots [101]. The study reported that out of 30 biomarkers, degradation of 27 biomarkers was primarily driven by high humidity, out of which 7 markers saw a 90% loss in concentration by the end of the study, with 4 of those markers losing over half their initial levels within just 1 week of storage. This highlights the critical need for immediate desiccation in home‐sampling kits and the use of heat‐resistant packaging [101]. Findings from another study indicate that cytokine levels are highly sensitive to sample handling; specifically, while IL‐6 remains stable in unseparated EDTA plasma for 24 h, unseparated serum requires immediate processing or refrigeration to prevent inaccurate results [102].

### 6.5 Analytical Sensitivity and Suitability for the Biomarker Assays

Identifying target analytes from a micro‐volume sample could be challenging owing to sensitivity issues. The physiological concentrations of biomarkers like alpha‐1 antitrypsin and C‐RP are high (mg/L or mg/dL) [103], and hence are easily detected in microvolume samples. Methods are available in the literature that have been validated for their measurement from microsamples [37]. However, biomarkers like interleukins exist in very low concentrations (often in the pg/mL range). Their measurements from microsamples require high‐sensitivity detection methods like multiplex immunoassay (Luminex/MSD) or LC–MS/MS‐based assays [104]. BNP and NT‐proBNP also exist in pg/mL concentrations. While traditionally requiring larger venous volumes, new chemiluminescence and photonic suspension assays have reached limits of detection (LOD) of as low as 4 pg/mL for BNP and 0.058 pg/mL for NT‐proBNP, making their detection from microvolume samples possible [105]. The clinical utility of microsampling for biomarker analysis will be defined by its ability to accurately quantify proteins across broad physiological ranges spanning pg/mL (such as BNP or NT‐proBNP) or mg/L (such as C‐RP). While this may require introduction and validation of sensitive analytical workflows such as LC‐MS/MS, clinical microsampling may be more amenable for other markers. For instance, distinguishing low‐risk C‐RP levels (< 1 mg/L) from acute inflammatory states (> 10 mg/L) [106], or identifying Alpha‐1 Antitrypsin (AAT) deficiency (< 50 mg/dL) versus normal physiological concentrations (100–273 mg/dL) [103]. The game‐changing nature of dried blood microsampling devices currently excludes whole‐cell analysis. Because standard microsampling relies on the drying of blood‐ a process that inevitably triggers cell lysis, it is currently unsuitable for applications requiring intact cellular morphology [107]. An example for biomarker suitability for microsampling is the quantification of biomarkers like periostin from microvolume samples. This remains technically challenging because clinical reference ranges are established using serum, and the microsampling devices primarily collect capillary whole blood. This necessitates complex validation to ensure that whole‐blood measurements accurately reflect established serum‐based reference ranges.

## 7 Conclusion and Future Directions

Blood micro‐sampling techniques are a novel and innovative means for collecting and analyzing micro‐volume blood samples for biomarker identification and quantification. These tools can open new avenues in respiratory research wherein treatment optimization as per the individual's biomarker profile can help clinicians achieve better control over the heterogeneity of respiratory diseases. The transition from traditional venous phlebotomy to patient‐centric microsampling represents a paradigm shift in respiratory medicine. As examined in this review, high‐value biomarkers for lung injury and inflammation, such as sRAGE, CCL‐18, and Surfactant Proteins A and D, possess the physiological concentrations and biochemical stability required for low‐volume analysis. While traditional studies relied on large‐volume draws to identify these markers, modern analytical techniques like high‐sensitivity ELISA and LC–MS/MS have proven that clinically actionable data can be extracted from as little as 20–50 μL of capillary blood. Additionally, the emergence of liquid‐matrix devices like the Tasso‐SST addresses a critical technical hurdle by providing high‐quality serum, thereby avoiding the extraction inefficiencies and “hematocrit bias” historically associated with dried blood spots (DBS). This capability ensures that large, complex glycoproteins essential for monitoring interstitial lung disease and COPD can be captured with high fidelity outside of a clinical setting. The successful application of novel microsampling tools to the biomarkers detailed in Table 1 would provide a highly tractable approach to advance the study of biomarkers of respiratory disease patients, particularly where validation would benefit from home‐based microsampling. By bringing sampling and analysis into the patient's home, the potential for high‐frequency, data‐rich biomarker monitoring provides substantial future rewards for respiratory disease diagnosis and management.

## Funding

Ahmar Khan was in receipt of an MSc studentship which was part‐funded by the Capitainer Ab, Hertfordshire Local Enterprise Partnership's “Growth Deal 2”, and the European Regional Development Fund (England) 17R18P02396.

## Conflicts of Interest

The authors declare no conflicts of interest.

## Supporting information


**Table S1:** Literature on the peer‐reviewed clinical evidence supporting respiratory biomarkers, along with the analytical methods and biological matrices used for their measurement.

**Table S2:** Summary of the published literature demonstrating the utilization of novel micro‐sampling devices for biomarker studies, including respiratory biomarkers, analytical platforms.
