# Supplementary material for: Respiratory Biomarkers: An Opportunity for Patient‐Centric Microsampling Approach for Treatment Optimization
Source: Clin Transl Sci. 2026 Jun 1;19(6):e70600. doi: 10.1111/cts.70600 (PMC13240354; doi:10.1111/cts.70600)
Supplement: Supplementary file 1 — Table S1: Literature on the peer‐reviewed clinical evidence supporting respiratory biomarkers, along with the analytical methods and biological matrices used for their measurement. [file CTS-19-e70600-s002.docx]

**Supplementary Data**

**Table S1. Literature on the peer-reviewed clinical evidence supporting respiratory biomarkers, along with the analytical methods and biological matrices used for their measurement.**

| Biomarker | Disease involved | Analytical approach and sample matrix | Peer-reviewed clinical evidence for use in clinical decision making |
| --- | --- | --- | --- |
| Eosinophils/ activation markers | COPD, AECOPD. | ELISA, flow cytometry, LC-MS/MS, Automated hematology analyzer [1–3]  **Sample matrix**- Whole blood, lung tissue. | Extensive clinical evidence demonstrates that blood eosinophil counts are highly predictive of a patient’s therapeutic response to inhaled corticosteroids (ICS) [4] and inhaled short-acting beta-2 agonists [5–7].  ***Clinical decision-*** *Therapeutic Selection*  ***Precision clinical outcome-*** *Predicts responsiveness to Inhaled Corticosteroids (ICS) in COPD and guides biologic choice (anti-IL5) in severe asthma [4,5,8].* |
| Neutrophils and their derivatives (Neutrophil elastase) | Asthma, COPD, Influenza, Bronchiectasis. | ELISA, LC-MS/MS, Flow cytometry [9–11]  **Sample matrix**- Whole blood, serum, plasma, sputum. | Persistent neutrophilic infiltration represents a hallmark of various chronic inflammatory lung conditions, most notably bronchiectasis, cystic fibrosis, asthma, and chronic obstructive pulmonary disease [12]. Point-of-care testing for neutrophil elastase (NE) could streamline its adoption as a clinical biomarker, directly informing real-time therapeutic strategies [12]. A retrospective study identified the Neutrophil/Lymphocyte Ratio as a marker of severe Influenza during the 2024–2025 outbreak in France [13].  ***Clinical decision-*** *Steroid responsiveness, Risk stratification*  ***Precision clinical outcome-*** *Indicates steroid-resistant phenotypes. In asthma, if a patient has high sputum neutrophils but low eosinophils, clinicians may decide not to escalate inhaled corticosteroids (ICS) and instead look for underlying infections or environmental triggers [14]. Additionally, guides the incorporation of macrolides for reducing the frequency of flare-ups in individuals suffering from chronic respiratory conditions [12].* |
| Immunoglobulin E (IgE) | Asthma, Cystic fibrosis, Allergic rhinitis. | Fluorescent Enzyme Immunoassay (FEIA), LC-MS/MS  **Sample matrix**- Whole blood, serum, plasma. | IgE is the definitive biomarker for phenotyping allergic airway diseases, guiding the selection and dosing of targeted biologics like omalizumab [15,16]. Longitudinal monitoring of IgE identifies the T2-high inflammatory trait, enabling "steroid-sparing" decisions that significantly reduce exacerbations and optimize long-term clinical outcomes [17].  ***Precision clinical outcome-*** *IgE levels are used to differentiate between airway diseases of allergic and non-allergic origin. Also, IgE levels indicate whether a patient is a candidate for anti-IgE therapy (Omalizumab/Xolair). Additionally, Omalizumab dosing is calculated using a precision medicine formula based on the patient's baseline Total IgE and body weight [15,16].* |
| Exosomes | COPD, Lung cancer, TB, ILD. | QTOF-LC-MS/MS, flow cytometry [18,19]  **Sample matrix**- Serum, plasma, saliva, urine | Studies have revealed that exosomes have a cardinal role to play in the pathogenesis of COPD, including the development of emphysema, chronic bronchitis [20,21], lung cancer, tuberculosis, and interstitial lung disease [22]. Extraction of exosomes from human plasma, urine, saliva, amniotic, and cerebrospinal fluids for global exosomal metabolome profiling has also been done [19].  ***Clinical decision-*** *Liquid Biopsy*  ***Precision clinical outcome-*** *Monitoring tumor-derived microRNA or DNA in exosomes to track lung cancer mutations and resistance to tyrosine kinase inhibitors* |
| Periostin | Asthma, Idiopathic Pulmonary Fibrosis (IPF) | ELISA, Electrochemiluminescence assay- Elecsys® Periostin assay (Roche Diagnostics, Penzberg, Germany), LC-MS/MS [23–25].  **Sample matrix**- Serum (gold standard), BALF, sputum. | Research has established periostin as a key biomarker for assessing eosinophilic inflammation, subepithelial fibrosis, and structural airway remodeling in various respiratory conditions [23,24,26]. Furthermore, emerging evidence indicates that monomeric periostin may offer superior sensitivity for monitoring Idiopathic Pulmonary Fibrosis (IPF) than total periostin, as the latter includes multimeric variants often linked to bone metabolism rather than lung pathology [27]. Furthermore, differential diagnosis relies on a combinatorial approach along with other markers like KL-6 for IPF, or FeNO and IgE for Th2- high asthma [28].  ***Clinical decision-*** *Phenotypic-driven biologic selection*  ***Precision clinical outcome-*** *Elevated serum periostin serves as a definitive marker for the T2-high phenotype, identifying patients with eosinophilic inflammation and structural airway thickening. These high levels are clinically used to predict superior therapeutic responses to IL-13 inhibitors (e.g., Lebrikizumab) and anti-IgE biologics (e.g., omalizumab), leading to significantly improved lung function outcomes [24–26,29].* |
| Alpha 1 antitrypsin | COPD | Immunoassay, LC-MS/MS, capillary electrophoresis [30–32].  **Sample matrix**- Serum, plasma, stool, lung tissue | Patients with severe-deficient genotypes account for severe AATD with clinically significant emphysema and need Alpha-1 Antitrypsin (AAT) augmentation therapy to prevent emphysema from advancing [33].Oxidized AAT was a biomarker for smoke-induced COPD and for evaluating the potential of antioxidant therapy in COPD [34]. Elevated levels of plasma desmosine and isodesmosine correlate with decreased lung function in AATD-induced COPD [35–38], including those with normal spirometry [39].  ***Clinical decision-*** *Disease Management*  ***Precision clinical outcome-*** *The identification of deficiency necessitates the implementation of augmentation therapy to prevent emphysema from advancing and calls for the screening of family member [33,40].* |
| Plasma fibrinogen | COPD comorbid with cardiovascular disease | clotting rate assays, pro-thrombin derived, ELISA [41].  **Sample matrix**- Plasma | Plasma fibrinogen is associated with coronary artery diseases, vascular and non-vascular mortality, stroke, and metabolic syndrome as well [42,43], and can identify co-morbidities associated with COPD.  ***Clinical decision-*** *Risk Stratification*  ***Precision clinical outcome-*** *Elevated levels identify COPD patients who are at increased risk for recurrent exacerbations, requiring more proactive preventive management. Additionally, elevated fibrinogen levels could be a major contributor to the development of co-morbidities like coronary artery disease associated with COPD [42–44].* |
| sRAGE | COPD, Covid-19 | ELISA, LC-MS/MS [45,46]  **Sample matrix**- Serum, plasma, Lung tissue | sRAGE is a promising blood biomarker for COPD when combined in a panel of other blood biomarkers [47], including progression of emphysema and reduced lung function [45,47–49]. The relationship to disease progression is dependent on the presence of genetic polymorphism [46–48].  ***Clinical decision-*** *Assessment of alveolar injury*  ***Precision clinical outcome-*** *Predicts the progression of emphysema and ARDS in high-risk patients. Also, differentiates between hypo-inflammatory and hyper-inflammatory sub phenotypes [45,50].* |
| C-RP | Systemic inflammation, COPD, CVD, Lung cancer | ELISA, LC-MS/MS, Nephelometry [51–53].  **Sample matrix**- Serum, plasma, sputum, lung tissue, exhaled breath condensate. | Elevated C-RP levels are linked to a higher risk of mortality and severe exacerbations in COPD patients [54], as well as identifying underlying inflammatory activity in smokers without the disease [55]. Studies have shown that antibiotic prescribing guided by C-RP levels during COPD exacerbations led to a reduction in both patient-reported antibiotic use and clinician-issued prescriptions, without any indication of adverse effects [56–58].  ***Clinical decision- Antimicrobial Stewardship***  ***Precision clinical outcome-*** *Utilised in primary care to effectively minimise unwarranted antibiotic prescriptions for acute exacerbations of COPD [56,57].* |
| IL-6 and IL-8 | Lung cancer, Covid-19, ILD | Electrochemiluminescence immunoassay (ECLIA), flow cytometry [59,60]  **Sample matrix**- Serum, plasma, sputum, nasal lavage. | High level of serum IL-6 in ILD could predict acute exacerbation and poor prognosis in patients with ILD [60], and predict COVID-19 severity [61], although IL-8 was a more sensitive biomarker for COVID-19 [61]. Serum IL-6 and IL-8 levels were potential diagnostic biomarkers for lung cancer [62].  ***Clinical decision- Immunomodulation, diagnosis and severity tracking***  ***Precision clinical outcome-*** *High levels of IL-6 identify patients with COVID-19 or ARDS who will benefit from IL-6 receptor antagonists like Tocilizumab. IL-8 acts as a marker of neutrophilic inflammation and mucus hypersecretion is used to monitor cystic fibrosis and bronchiectasis [63–65].* |
| Serum surfactant proteins- A and D | COPD, COVID-19, COVID-19-like pneumonia, RDS, IPF, cystic fibrosis. | ELISA, chemiluminescent enzyme immunoassay (CLEIA) [66,67]  **Sample matrix**- Serum, plasma, lung tissue. | Association between elevated serum SP-D levels and increased severity of COVID-19 [66]. Lower serum levels of SP-D and Krebs von den Lungen (KL-6) could distinguish COVID-19 pneumonia from that of COVID-19 pneumonia-like disease [67].  ***Clinical decision-*** *Barrier integrity*  ***Precision clinical outcome-*** *Elevated serum levels indicate damage to the alveolar-capillary barrier, specifically in Idiopathic pulmonary fibrosis (IPF) [68,69].* |
| Brain Natriuretic peptide (BNP) and NT-pro BNP | COPD comorbid with CVD | LC-MS/MS [70–72]. Immunoassays (e.g. TriageBNP, ADVIA Centaur BNP®, AxSYM® BNP assay) [73]  **Sample matrix**- Serum, plasma. | Elevation of serum BNP and NT proBNP levels in COPD patients confirmed by metaanalysis [74], and in pulmonary hypertension in COPD patients [75]. Increase in the BNP/NT proBNP occurs during COPD exacerbation offering differential diagnostic biomarker for acute dyspnea of cardiac or pulmonary origin [74] and decide optimum therapy [76]. BNP testing improved the detection of chronic heart failure in COPD patients by 20%, including in patients with no history of heart failure [77].  ***Clinical decision-*** *Differential Diagnosis*  ***Precision clinical outcome-*** *Quickly distinguishes acute heart failure from pulmonary origins (Asthma/COPD) in patients with acute dyspnoea. Serum levels of both BNP and NT-proBNP can also be utilized as a predictive biomarker in pulmonary hypertension [74,76].* |
| CCL-18 | COPD | ELISA [78]  **Sample matrix**- Serum, plasma, lung tissue. | Serum concentrations of CCL-18 were significantly higher in COPD patients than in healthy individuals, and that there was a correlation between higher levels of CCL-18 in serum and the number of COPD exacerbations [78] . Serum CCL-18 level was predictive for the application of inhaled corticosteroids in hospitalized COPD patients [78].  ***Clinical decision-*** *Prognostication*  ***Precision clinical outcome-*** *Elevated serum level is among the most dependable indicators of mortality and lung function deterioration in IPF [79–81].* |
| TNF-alpha | COPD, asthma, ARDS, sarcoidosis, and ILD | Immunoassays, Flow cytometry, ELISA [82].  **Sample matrix**- Serum, plasma, sputum, exhaled breath condensate. | A meta-analysis study found that serum TNFα levels were significantly higher in the COPD group than in the control group [83], and both TNF-α and interleukin (IL-1β) were elevated in patients with COPD, highlighting their role as biomarkers for COPD and the severity of airflow limitation [84].  ***Clinical decision-*** *Disease phenotyping*  ***Precision clinical outcome-****Identifies "High-Inflammatory" phenotypes at risk for rapid FEV_1_ decline and frequent hospitalizations [85].* |
| Pro calcitonin (PCT) | Community-acquired pneumonia (CAP) and other bacterial respiratory infections. | TRACE (Time-resolved amplified cryptate emission technology assay (Kryptor PCT; Brahms AG,) [86,87]  **Sample matrix**- Serum, plasma. | PCT has been widely used to guide antibiotic treatment [87–89] and PCT assays has been approved by the USA Food and Drug Administration to guide antibiotic treatment in respiratory tract infections [86,90]. A meta-analysis study showed PCT-based antibiotic treatment lowers antibiotic exposure and side effects, and improved survival [91], while PCT has shown potential to distinguish between bacterial or viral respiratory infections [92].  ***Clinical decision-*** *Infection Control*  ***Precision clinical outcome-*** *Guiding the initiation and more importantly the duration of antibiotic therapy in lower respiratory tract infections [90,93].* |

**References.**

1. **Ochkur SI, Kim JD, Protheroe CA, *et al.*** A sensitive high throughput ELISA for human eosinophil peroxidase: A specific assay to quantify eosinophil degranulation from patient-derived sources. *Journal of Immunological Methods* 2012; **384**(1–2): 10–20. doi: 10.1016/j.jim.2012.06.011.

2. **Thomas ME, Markowitz JE, Arwood AC, Germany JM, Gilliland WM**. An LC-MS/MS method for the quantification of 3-bromotyrosine in plasma from patients diagnosed with eosinophilic esophagitis. *Analytical Methods* 2024; **16**(38): 6509–6516. doi: 10.1039/D4AY00697F.

3. **Ethier C, Lacy P, Davoine F**. *Identification of Human Eosinophils in Whole Blood by Flow Cytometry*. 2014:81–92.

4. **Xie C, Wang K, Yang K, *et al.*** Toward precision medicine in COPD: phenotypes, endotypes, biomarkers, and treatable traits. *Respiratory Research* 2025; **26**(1): 274. doi: 10.1186/s12931-025-03356-w.

5. **Vanetti  Marco, Visca  Dina, Ardesi  Francesco, Zappa  Martina, Pignatti  Patrizia, Spanevello  Antonio**. Eosinophils in chronic obstructive pulmonary disease. *Therapeutic Advances in Respiratory Disease* SAGE Publications Ltd STM, 2025; **19**: 17534666251335800. doi: 10.1177/17534666251335800.

6. **Lupia C, Pastore D, Marrazzo G, *et al.*** Short-Term Effects of Dupilumab in Eosinophilic COPD. *Journal of Clinical Medicine* 2026; **15**(2): 775. doi: 10.3390/jcm15020775.

7. **Komura M, Sato T, Suzuki Y, *et al.*** Blood Eosinophil Count as a Predictive Biomarker of Chronic Obstructive Pulmonary Disease Exacerbation in a Real-World Setting. *Canadian Respiratory Journal* 2023; **2023**: 1–8. doi: 10.1155/2023/3302405.

8. **David B, Bafadhel M, Koenderman L, De Soyza A**. Eosinophilic inflammation in COPD: from an inflammatory marker to a treatable trait. *Thorax* 2021; **76**(2): 188. doi: 10.1136/thoraxjnl-2020-215167.

9. **Sadiku P, Brenes AJ, Mayer RL, *et al.*** Single cell proteomic analysis defines discrete neutrophil functional states in human glioblastoma. *Nature Communications* 2025; **17**(1): 621. doi: 10.1038/s41467-025-67367-3.

10. **Brunck MEG, Andersen SB, Timmins NE, Osborne GW, Nielsen LK**. Absolute counting of neutrophils in whole blood using flow cytometry. *Cytometry Part A* 2014; **85**(12): 1057–1064. doi: 10.1002/cyto.a.22503.

11. **Stoimenou M, Tzoros G, Skendros P, Chrysanthopoulou A**. Methods for the Assessment of NET Formation: From Neutrophil Biology to Translational Research. *International journal of molecular sciences* 2022; **23**(24): . doi: 10.3390/ijms232415823.

12. **Chalmers JD, Mall MA, Nielsen KG, *et al.*** Neutrophil-derived biomarkers in bronchiectasis: identifying a common therapeutic target. *European Respiratory Journal* 2025; **66**(3): 2500081. doi: 10.1183/13993003.00081-2025.

13. **Vassallo M, Derollez M, Veaute M-H, *et al.*** The Neutrophil/Lymphocyte Ratio Was Identified as a Marker of Severe Influenza During the 2024–2025 Outbreak in France. *Infectious Disease Reports* 2025; **17**(5): 127. doi: 10.3390/idr17050127.

14. **Crisford H, Sapey E, Rogers GB, *et al.*** Neutrophils in asthma: the good, the bad and the bacteria. *Thorax* 2021; **76**(8): 835–844. doi: 10.1136/thoraxjnl-2020-215986.

15. **Gevaert P, Wong K, Millette LA, Carr TF**. The Role of IgE in Upper and Lower Airway Disease: More Than Just Allergy! *Clinical reviews in allergy & immunology* 2022; **62**(1): 200–215. doi: 10.1007/s12016-021-08901-1.

16. ***GINA guidelines 2025***.

17. **Sousa‐Pinto B, Bousquet J, Vieira RJ, *et al.*** Allergic Rhinitis and Its Impact on Asthma ( <scp>ARIA</scp> )‐ <scp>EAACI</scp> Guidelines—2024–2025 Revision: Part I—Guidelines on Intranasal Treatments. *Allergy* 2025; . doi: 10.1111/all.70131.

18. **Garza AP, Wider-Eberspächer E, Morton L, *et al.*** Proteomic analysis of plasma-derived extracellular vesicles: pre- and postprandial comparisons. *Scientific Reports* 2024; **14**(1): 23032. doi: 10.1038/s41598-024-74228-4.

19. **Lu F, Cheng X, Qi X, Li D, Hu L**. Metabolic landscaping of extracellular vesicles from body fluids by phosphatidylserine imprinted polymer enrichment and mass spectrometry analysis. *Talanta* 2025; **282**: 126940. doi: 10.1016/j.talanta.2024.126940.

20. **Wang N, Wang Q, Du T, *et al.*** The Potential Roles of Exosomes in Chronic Obstructive Pulmonary Disease. *Frontiers in Medicine* 2021; **7**: . doi: 10.3389/fmed.2020.618506.

21. **Mahboub B, Hamoudi R, Hachim MY, Busch H**. Editorial: Biomarkers in Pulmonary Diseases. *Frontiers in Medicine* 2022; **9**: . doi: 10.3389/fmed.2022.790475.

22. **Fu J, Song W, Hao Z, Fan M, Li Y**. Research trends and hotspots of exosomes in respiratory diseases. *Medicine* 2023; **102**(39): e35381. doi: 10.1097/MD.0000000000035381.

23. **Rusbjerg-Weberskov CE, Gant MS, Chamot-Rooke J, Nielsen NS, Enghild JJ**. Development of a top-down MS assay for specific identification of human periostin isoforms. *Frontiers in Molecular Biosciences* 2024; **11**: . doi: 10.3389/fmolb.2024.1399225.

24. **Fingleton J, Braithwaite I, Travers J, *et al.*** Serum periostin in obstructive airways disease. *European Respiratory Journal* 2016; **47**(5): 1383–1391. doi: 10.1183/13993003.01384-2015.

25. **Pavlidis S, Takahashi K, Ng Kee Kwong F, *et al.*** “T2-high” in severe asthma related to blood eosinophil, exhaled nitric oxide and serum periostin. *European Respiratory Journal* 2019; **53**(1): 1800938. doi: 10.1183/13993003.00938-2018.

26. **Ravindran S, Kaleem Ullah M, Karnik M, *et al.*** Unsupervised Phenotyping of Asthma: Integrating Serum Periostin with Clinical and Inflammatory Profiles. *Diagnostics* 2025; **15**(23): 3028. doi: 10.3390/diagnostics15233028.

27. **Ohta S, Okamoto M, Fujimoto K, *et al.*** The usefulness of monomeric periostin as a biomarker for idiopathic pulmonary fibrosis. *PLOS ONE* 2017; **12**(3): e0174547. doi: 10.1371/journal.pone.0174547.

28. **Izuhara K, Conway SJ, Moore BB, *et al.*** Roles of Periostin in Respiratory Disorders. *American Journal of Respiratory and Critical Care Medicine* 2016; **193**(9): 949–956. doi: 10.1164/rccm.201510-2032PP.

29. **Izuhara K, Ohta S, Ono J**. Using Periostin as a Biomarker in the Treatment of Asthma. *Allergy, Asthma & Immunology Research* 2016; **8**(6): 491. doi: 10.4168/aair.2016.8.6.491.

30. **Starcher B, Green M, Scott M**. Measurement of Urinary Desmosine as an Indicator of Acute Pulmonary Disease. *Respiration* 1995; **62**(5): 252–257. doi: 10.1159/000196458.

31. **Ma S, Turino GM, Lin YY**. Quantitation of desmosine and isodesmosine in urine, plasma, and sputum by LC–MS/MS as biomarkers for elastin degradation. *Journal of Chromatography B* 2011; **879**(21): 1893–1898. doi: 10.1016/j.jchromb.2011.05.011.

32. **Annovazzi L, Viglio S, Perani E, *et al.*** Capillary electrophoresis with laser‐induced fluorescence detection as a novel sensitive approach for the analysis of desmosines in real samples. *ELECTROPHORESIS* 2004; **25**(4–5): 683–691. doi: 10.1002/elps.200305607.

33. **Serban KA, Pratte KA, Strange C, *et al.*** Unique and shared systemic biomarkers for emphysema in Alpha-1 Antitrypsin deficiency and chronic obstructive pulmonary disease. *eBioMedicine* 2022; **84**: 104262. doi: 10.1016/j.ebiom.2022.104262.

34. **Topic A, Milovanovic V, Lazic Z, Ivosevic A, Radojkovic D**. Oxidized Alpha-1-Antitrypsin as a Potential Biomarker Associated with Onset and Severity of Chronic Obstructive Pulmonary Disease in Adult Population. *COPD: Journal of Chronic Obstructive Pulmonary Disease* 2018; **15**(5): 472–478. doi: 10.1080/15412555.2018.1541448.

35. **Huang JT-J, Chaudhuri R, Albarbarawi O, *et al.*** Clinical validity of plasma and urinary desmosine as biomarkers for chronic obstructive pulmonary disease. *Thorax* 2012; **67**(6): 502–508. doi: 10.1136/thoraxjnl-2011-200279.

36. **Lindberg CA, Engström G, de Verdier MG, *et al.*** Total desmosines in plasma and urine correlate with lung function. *European Respiratory Journal* 2012; **39**(4): 839–845. doi: 10.1183/09031936.00064611.

37. **Janoff A, Chanana AD, Joel DD, *et al.*** Evaluation of the Urinary Desmosine Radioimmunoassay as a Monitor of Lung Injury after Endobronchial Elastase Instillation in Sheep. *American Review of Respiratory Disease* 1983; **128**(3): 545–551. doi: 10.1164/arrd.1983.128.3.545.

38. **Stone PJ, Bryan-Rhadfi J, Lucey EC, *et al.*** Measurement of Urinary Desmosine by Isotope Dilution and High Performance Liquid Chromatography: Correlation between Elastase-induced Air-Space Enlargement in the Hamster and Elevation of Urinary Desmosine. *American Review of Respiratory Disease* 1991; **144**(2): 284–290. doi: 10.1164/ajrccm/144.2.284.

39. **Beiko T, Ma S, Strange C, M Turino G**. Biomarker indicators of elastin degradation in asymptomatic alpha-1 antitrypsin deficiency. *Pulmonary and Critical Care Medicine* 2018; **3**(3): . doi: 10.15761/PCCM.1000155.

40. **Stoller JK, Aboussouan LS**. α1-antitrypsin deficiency. *The Lancet* 2005; **365**(9478): 2225–2236. doi: 10.1016/S0140-6736(05)66781-5.

41. **Li C, Sun Z, Liu Y, Zhou W, Wang Y, Peng M**. Comparison among different measurement systems for fibrinogen using fresh samples and frozen samples. *Clinica Chimica Acta* 2020; **509**: 258–263. doi: 10.1016/j.cca.2020.06.030.

42. **Danesh J, Lewington S, Thompson SG et al.** *Plasma Fibrinogen Level and the Risk of Major Cardiovascular Diseases and Nonvascular Mortality An Individual Participant Meta-analysis*. 2005.

43. **Ford ES**. The metabolic syndrome and C-reactive protein, fibrinogen, and leukocyte count: findings from the Third National Health and Nutrition Examination Survey. *Atherosclerosis* Elsevier, 2003; **168**(2): 351–358. doi: 10.1016/S0021-9150(03)00134-5.

44. **Mohan M, Parthasarathi A, S K C, Biligere Siddaiah J, Mahesh PA**. Fibrinogen: A Feasible Biomarker in Identifying the Severity and Acute Exacerbation of Chronic Obstructive Pulmonary Disease. *Cureus* 2021; . doi: 10.7759/cureus.16864.

45. **Erusalimsky JD**. The use of the soluble receptor for advanced glycation-end products (sRAGE) as a potential biomarker of disease risk and adverse outcomes. *Redox Biology* 2021; **42**: 101958. doi: 10.1016/j.redox.2021.101958.

46. **Klont F, Joosten MR, Ten Hacken NHT, Horvatovich P, Bischoff R**. Quantification of the soluble Receptor of Advanced Glycation End-Products (sRAGE) by LC-MS after enrichment by strong cation exchange (SCX) solid-phase extraction (SPE) at the protein level. *Analytica Chimica Acta* 2018; **1043**: 45–51. doi: 10.1016/j.aca.2018.09.050.

47. **Wiersma VR, Pouwels SD**. An Integrative Genomic Strategy Identifies Soluble Receptor for Advanced Glycation End-Products as a Causal and Protective Biomarker of Lung Function. *CHEST* 2022; **161**(1): 3–5. doi: 10.1016/j.chest.2021.08.005.

48. **Pratte KA, Curtis JL, Kechris K, *et al.*** Soluble receptor for advanced glycation end products (sRAGE) as a biomarker of COPD. *Respiratory Research* 2021; **22**(1): 127. doi: 10.1186/s12931-021-01686-z.

49. **Klont F, Horvatovich P, Bowler RP, *et al.*** Plasma sRAGE levels strongly associate with centrilobular emphysema assessed by HRCT scans. *Respiratory Research* 2022; **23**(1): 15. doi: 10.1186/s12931-022-01934-w.

50. **Calfee CS, Ware LB, Eisner MD, *et al.*** Plasma receptor for advanced glycation end products and clinical outcomes in acute lung injury. *Thorax* 2008; **63**(12): 1083–1089. doi: 10.1136/thx.2008.095588.

51. **Orens P, Lame M, Calciano S, Chambers E**. *Sensitive and Reproducible LC-MS Quantification of C-Reactive Protein in Plasma: A Potential Biomarker of Inflammation*.

52. **Zhang L, Li H-Y, Li W, *et al.*** An ELISA Assay for Quantifying Monomeric C-Reactive Protein in Plasma. *Frontiers in Immunology* 2018; **9**: . doi: 10.3389/fimmu.2018.00511.

53. **Drieghe SA, Alsaadi H, Tugirimana PL, Delanghe JR**. A new high-sensitive nephelometric method for assaying serum C-reactive protein based on phosphocholine interaction. *Clinical Chemistry and Laboratory Medicine (CCLM)* 2014; **52**(6): . doi: 10.1515/cclm-2013-0669.

54. **Oshagbemi OA, Franssen FME, Wouters EFM, *et al.*** C-reactive protein as a biomarker of response to inhaled corticosteroids among patients with COPD. *Pulmonary Pharmacology & Therapeutics* Academic Press, 2020; **60**: 101870. doi: 10.1016/J.PUPT.2019.101870.

55. **Hassan A, Jabbar N**. C-reactive Protein as a Predictor of Severity in Chronic Obstructive Pulmonary Disease: An Experience From a Tertiary Care Hospital. *Cureus* 2022; . doi: 10.7759/cureus.28229.

56. **Prins HJ, Duijkers R, van der Valk P, *et al.*** CRP-guided antibiotic treatment in acute exacerbations of COPD in hospital admissions. *European Respiratory Journal* 2019; **53**(5): 1802014. doi: 10.1183/13993003.02014-2018.

57. **Butler CC, Gillespie D, White P, *et al.*** C-Reactive Protein Testing to Guide Antibiotic Prescribing for COPD Exacerbations. *New England Journal of Medicine* 2019; **381**(2): 111–120. doi: 10.1056/NEJMoa1803185.

58. **de Torres JP, Cordoba-Lanus E, López-Aguilar C, *et al.*** C-reactive protein levels and clinically important predictive outcomes in stable COPD patients. *European Respiratory Journal* 2006; **27**(5): 902–907. doi: 10.1183/09031936.06.00109605.

59. **Yan X, Han L, Zhao R, Fatima S, Zhao L, Gao F**. Prognosis value of IL-6, IL-8, and IL-1β in serum of patients with lung cancer: A fresh look at interleukins as a biomarker. *Heliyon* 2022; **8**(8): e09953. doi: 10.1016/j.heliyon.2022.e09953.

60. **Lee JH, Jang JH, Park JH, *et al.*** The role of interleukin-6 as a prognostic biomarker for predicting acute exacerbation in interstitial lung diseases. *PLOS ONE* 2021; **16**(7): e0255365. doi: 10.1371/journal.pone.0255365.

61. **Li L, Li J, Gao M, *et al.*** Interleukin-8 as a Biomarker for Disease Prognosis of Coronavirus Disease-2019 Patients. *Frontiers in Immunology* 2021; **11**: . doi: 10.3389/fimmu.2020.602395.

62. **Yan X, Han L, Zhao R, Fatima S, Zhao L, Gao F**. Prognosis value of IL-6, IL-8, and IL-1β in serum of patients with lung cancer: A fresh look at interleukins as a biomarker. *Heliyon* 2022; **8**(8): e09953. doi: 10.1016/j.heliyon.2022.e09953.

63. **Abani O, Abbas A, Abbas F, *et al.*** Tocilizumab in patients admitted to hospital with COVID-19 (RECOVERY): a randomised, controlled, open-label, platform trial. *The Lancet* Elsevier, 2021; **397**(10285): 1637–1645. doi: 10.1016/S0140-6736(21)00676-0.

64. **Cesta MC, Zippoli M, Marsiglia C, *et al.*** The Role of Interleukin-8 in Lung Inflammation and Injury: Implications for the Management of COVID-19 and Hyperinflammatory Acute Respiratory Distress Syndrome. *Frontiers in Pharmacology* 2022; **12**: . doi: 10.3389/fphar.2021.808797.

65. **Angel A Justiz Vaillant AQurie**. *Interleukin*. StatPearls Publishing, 2022.

66. **Tong M, Xiong Y, Zhu C, *et al.*** Serum surfactant protein D in COVID-19 is elevated and correlated with disease severity. *BMC Infectious Diseases* 2021; **21**(1): 737. doi: 10.1186/s12879-021-06447-3.

67. **Togashi Y, Kono Y, Okuma T, *et al.*** Surfactant protein D: A useful biomarker for distinguishing COVID‐19 pneumonia from COVID‐19 pneumonia‐like diseases. *Health Science Reports* 2022; **5**(3): . doi: 10.1002/hsr2.622.

68. **Deng Y-P, Sun J, He Q-Y, Liu Y, Fu L, Zhao H**. The value of surfactant protein a in evaluating the severity and prognosis in community-acquired pneumonia patients. *BMC Pulmonary Medicine* 2024; **24**(1): 472. doi: 10.1186/s12890-024-03297-y.

69. **Greene KE, King TE, Kuroki Y, *et al.*** Serum surfactant proteins‐A and ‐D as biomarkers in idiopathic pulmonary fibrosis. *European Respiratory Journal* 2002; **19**(3): 439–446. doi: 10.1183/09031936.02.00081102.

70. **Berna M, Ott L, Engle S, Watson D, Solter P, Ackermann B**. Quantification of NTproBNP in Rat Serum Using Immunoprecipitation and LC/MS/MS:  a Biomarker of Drug-Induced Cardiac Hypertrophy. *Analytical Chemistry* 2008; **80**(3): 561–566. doi: 10.1021/ac702311m.

71. **Torma AF, Groves K, Biesenbruch S, *et al.*** A candidate liquid chromatography mass spectrometry reference method for the quantification of the cardiac marker 1-32 B-type natriuretic peptide. *Clinical Chemistry and Laboratory Medicine (CCLM)* 2017; **55**(9): . doi: 10.1515/cclm-2016-1054.

72. **Chappell DL, Lee AY, Bernstein HS, Lassman ME, Laterza OF**. Development and Validation of an IA-LC/MS Method to Quantitate Active and Total B-Type Natriuretic Peptide in Human Plasma. *Bioanalysis* 2016; **8**(22): 2341–2349. doi: 10.4155/bio-2016-0195.

73. **Mayo DD, Colletti JE, Kuo DC**. Brain natriuretic peptide (BNP) testing in the emergency department. *The Journal of Emergency Medicine* 2006; **31**(2): 201–210. doi: 10.1016/j.jemermed.2005.08.022.

74. **Nicolae B, Ecaterina L**. Natriuretic peptides in elderly patients with chronic obstructive pulmonary disease. *The Egyptian Journal of Bronchology* 2022; **16**(1): 26. doi: 10.1186/s43168-022-00132-y.

75. **Vanderheyden M, Bartunek J, Goethals M**. Brain and other natriuretic peptides: molecular aspects. *European Journal of Heart Failure* 2004; **6**(3): 261–268. doi: 10.1016/j.ejheart.2004.01.004.

76. **Tian F, Song W, Wang L, *et al.*** NT-pro BNP in AECOPD-PH: old biomarker, new insights-based on a large retrospective case-controlled study. *Respiratory Research* 2021; **22**(1): 321. doi: 10.1186/s12931-021-01917-3.

77. **Calzetta L, Orlandi A, Page C, *et al.*** Brain natriuretic peptide: Much more than a biomarker. *International Journal of Cardiology* 2016; **221**: 1031–1038. doi: 10.1016/j.ijcard.2016.07.109.

78. **Dilektasli AG, Demirdogen Cetinoglu E, Uzaslan E, *et al.*** Serum CCL-18 level is a risk factor for COPD exacerbations requiring hospitalization. *International Journal of Chronic Obstructive Pulmonary Disease* 2017; **Volume 12**: 199–208. doi: 10.2147/COPD.S118424.

79. **Prasse A, Probst C, Bargagli E, *et al.*** Serum CC-Chemokine Ligand 18 Concentration Predicts Outcome in Idiopathic Pulmonary Fibrosis. *American Journal of Respiratory and Critical Care Medicine* 2009; **179**(8): 717–723. doi: 10.1164/rccm.200808-1201OC.

80. **Rong B, Fu T, Rong C, Liu W, Li K, Liu H**. Association between serum CCL-18 and IL-23 concentrations and disease progression of chronic obstructive pulmonary disease. *Scientific Reports* 2020; **10**(1): 17756. doi: 10.1038/s41598-020-73903-6.

81. **Sin DD, Miller BE, Duvoix A, *et al.*** Serum PARC/CCL-18 Concentrations and Health Outcomes in Chronic Obstructive Pulmonary Disease. *American Journal of Respiratory and Critical Care Medicine* 2011; **183**(9): 1187–1192. doi: 10.1164/rccm.201008-1220OC.

82. **Valaperti A, Li Z, Vonow-Eisenring M, Probst-Müller E**. Diagnostic methods for the measurement of human TNF-alpha in clinical laboratory. *Journal of Pharmaceutical and Biomedical Analysis* 2020; **179**: 113010. doi: 10.1016/j.jpba.2019.113010.

83. **Yao Y, Zhou J, Diao X, Wang S**. Association between tumor necrosis factor-α and chronic obstructive pulmonary disease: a systematic review and meta-analysis. *Therapeutic Advances in Respiratory Disease* 2019; **13**: . doi: 10.1177/1753466619866096.

84. **Shyam Prasad Shetty B, Chaya SK, Kumar V S, *et al.*** Inflammatory Biomarkers Interleukin 1 Beta (IL-1β) and Tumour Necrosis Factor Alpha (TNF-α) Are Differentially Elevated in Tobacco Smoke Associated COPD and Biomass Smoke Associated COPD. *Toxics* 2021; **9**(4): 72. doi: 10.3390/toxics9040072.

85. **Agustí A, Edwards LD, Rennard SI, *et al.*** Persistent Systemic Inflammation is Associated with Poor Clinical Outcomes in COPD: A Novel Phenotype. *PLoS ONE* 2012; **7**(5): e37483. doi: 10.1371/journal.pone.0037483.

86. **Huang DT, Yealy DM, Filbin MR, *et al.*** Procalcitonin-Guided Use of Antibiotics for Lower Respiratory Tract Infection. *New England Journal of Medicine* 2018; **379**(3): 236–249. doi: 10.1056/NEJMoa1802670.

87. **Christ-Crain M, Jaccard-Stolz D, Bingisser R, *et al.*** Effect of procalcitonin-guided treatment on antibiotic use and outcome in lower respiratory tract infections: cluster-randomised, single-blinded intervention trial. *The Lancet* 2004; **363**(9409): 600–607. doi: 10.1016/S0140-6736(04)15591-8.

88. **Christ-Crain M, Stolz D, Bingisser R, *et al.*** Procalcitonin Guidance of Antibiotic Therapy in Community-acquired Pneumonia. *American Journal of Respiratory and Critical Care Medicine* 2006; **174**(1): 84–93. doi: 10.1164/rccm.200512-1922OC.

89. **Schuetz P, Christ-Crain M, Thomann R, *et al.*** Effect of Procalcitonin-Based Guidelines vs Standard Guidelines on Antibiotic Use in Lower Respiratory Tract Infections. *JAMA* 2009; **302**(10): 1059. doi: 10.1001/jama.2009.1297.

90. **Creamer AW, Kent AE, Albur M**. Procalcitonin in respiratory disease: use as a biomarker for diagnosis and guiding antibiotic therapy. *Breathe* 2019; **15**(4): 296–304. doi: 10.1183/20734735.0258-2019.

91. **Schuetz P, Wirz Y, Sager R, *et al.*** Effect of procalcitonin-guided antibiotic treatment on mortality in acute respiratory infections: a patient level meta-analysis. *The Lancet Infectious Diseases* Elsevier, 2018; **18**(1): 95–107. doi: 10.1016/S1473-3099(17)30592-3.

92. **Sivapalan P, Jensen J-U**. Biomarkers in Chronic Obstructive Pulmonary Disease: Emerging Roles of Eosinophils and Procalcitonin. *Journal of Innate Immunity* 2022; **14**(2): 89–97. doi: 10.1159/000517161.

93. **Schuetz P, Wirz Y, Sager R, *et al.*** Effect of procalcitonin-guided antibiotic treatment on mortality in acute respiratory infections: a patient level meta-analysis. *The Lancet Infectious Diseases* 2018; **18**(1): 95–107. doi: 10.1016/S1473-3099(17)30592-3.
