# Supplementary material for: Respiratory Biomarkers: An Opportunity for Patient‐Centric Microsampling Approach for Treatment Optimization
Source: Clin Transl Sci. 2026 Jun 1;19(6):e70600. doi: 10.1111/cts.70600 (PMC13240354; doi:10.1111/cts.70600)
Supplement: Supplementary file 2 — Table S2: Summary of the published literature demonstrating the utilization of novel micro‐sampling devices for biomarker studies, including respiratory biomarkers, analytical platforms. [file CTS-19-e70600-s001.docx]

Table S2. Summary of the published literature demonstrating the utilization of novel micro-sampling devices for biomarker studies, including respiratory biomarkers, analytical platforms, and the choice of matrix.

| Title of the study | Biomarker(s) | Micro-sampling device | Assay platform | Biological matrix | Year |
| --- | --- | --- | --- | --- | --- |
| Development of a laboratory test on dried blood spots for facilitating early diagnosis of alpha-1-antitrypsin deficiency [1] | **alpha-1-antitrypsin** | Dried blood spot cards | Immunoturbidimetry and Immunonephelometry | Whole blood | 2014 |
| Detection of sulfur mustard-albumin adducts for poisoning confirmation [2] | sulfur mustard-albumin adducts | Mitra® device,  Noviplex DUO cards | LC-MS/MS | Plasma | 2016 |
| Quantitation of Estetrol [3] | Estetrol | Mitra® device | LC-MS/MS | Whole blood | 2017 |
| Isotopic analysis of iron in blood [4] | iron | Mitra® device | Multi-collector inductively coupled plasma-mass spectrometry (MC-ICP-MS) | Whole blood | 2017 |
| Measurement of C-peptide in whole blood [5] | C-peptide | DBS cards, Mitra® device | ELISA/Chemiluminescence assay | Whole blood | 2018 |
| HbA1C monitoring in diabetic children [6] | HbA1C | Mitra® device | HbA1c Tosoh G8 HPLC analysis | Whole blood | 2018 |
| Measurement of cortisol and testosterone in athletes [7] | cortisol and testosterone | Mitra® device | LC-MS/MS | Whole blood | 2018 |
| Determination of Vancomycin and Creatinine [8] | Creatinine | Mitra® device | LC-MS/MS | Plasma | 2019 |
| Measurement of  multidimensional anti-influenza IgG antibodies [9] | anti-influenza IgG antibodies | Mitra® device | mPlex-Flu assay | Whole blood | 2019 |
| Determination of iohexol for assessing glomerular filtration rate. [10] | iohexol | Mitra® device,  HemaPEN | UHPLC-MS/MS | Whole blood | 2019 |
| Fatty acid quantitation [11] | Fatty acids | Mitra® device,  903 Protein saver cards. | UHPLC-MS,  Gas chromatography (GC0 | Whole blood | 2019 |
| Quantification of intact IGF-I for antidoping analysis [12] | IGF-I | Mitra® device | LC-HRMS | Whole blood | 2020 |
| Determination of Hg [13] | Hg | Mitra® device,  Capitainer® B10,  HemaXis DB 10 | Atomic absorption spectrometry | Whole blood/ Urine | 2020 |
| Quantification of testosterone, androstenedione and 17-hydroxyprogesterone [14] | testosterone, androstenedione and 17-hydroxyprogesterone | Mitra® device | LC-MS/MS | Whole blood | 2020 |
| Metabolic profile monitoring during physical exercise [15] | Metabolites | hemaPEN | UHPLC-MS/MS | Whole blood | 2021 |
| Measurement of the alcohol biomarker phosphatidylethanol (PEth) [16] | phosphatidylethanol (PEth) | Mitra® device,  Capitainer® B10,  HemaXis DB 10, Whatman 903 Protein Saver card. | LC-MS/MS | Whole blood | 2021 |
| Untargeted lipidomics [17] | Lipids | Mitra® device | QTOF- LC-MS/MS | Whole blood | 2021 |
| Determination of Cu in blood [18] | Copper | Mitra® device,  Capitainer® B10,  Hemaxis DB 10, HemaPEN. | High-Resolution Continuum Source Graphite Furnace Atomic Absorption Spectrometry (HR CS GFAAS) | Whole blood | 2021 |
| Evaluation of the post-vaccination level of neutralizing antibodies against SARS-CoV-2 [19] | antibodies | Capitainer® B10 | Electrochemiluminescence immunoassay (ECLIA) Elecsys® (Roche Diagnostics) | Whole blood | 2021 |
| Evaluation of haemoglobin related clinical markers [20] | Hb markers | Mitra® device | LC-MS/MS | Whole blood | 2022 |
| Measurement of tacrolimus and creatinine after renal transplantation [21] | Creatine and Tacrolimus | Mitra® device, Whatman 903 card | LC-MS/MS | Whole blood | 2022 |
| Determination of Tryptophan-related biomarkers [22] | Tryptophan markers | Mitra® device | LC-MS/MS | Whole blood | 2022 |
| Assessing the use of a micro-sampling device for measuring blood protein levels in healthy subjects and COVID-19 patients [23] | PCT, IL-6, RAGE | Tasso-SST | Ella multi-analyte immuno-assay (ProteinSimple, San Jose, CA, USA) | Serum | 2022 |
| Measurement of phenylalanine and tyrosine to monitor patients with phenylketonuria [24] | phenylalanine and tyrosine | Mitra® device, Capitainer® B10, HemaXis B10, Bioanalysis RUO cards, PerkinElmer-226 filter paper collection devices | Flow injection analysis tandem mass spectrometry (FIA-MS/MS) | Whole blood | 2022 |
| Therapeutic drug monitoring of immunosuppressants and creatinine [25] | Creatinine and immunosuppresants | Capitainer® B10 | LC-MS/MS | Whole blood | 2023 |
| Measurement of Globotriaosylsphingosine and Its Analogues [26] | Globotriaosylsphingosine | Capitainer® B10,  Whatman 903 cards | UHPLC-MS/MS | Whole blood | 2023 |
| Determination of ceramides biomarkers [27] | Ceramide biomarkers | Capitainer® B10 | UHPLC-MS/MS | Whole blood | 2023 |
| Targeted lipidomic study to monitor 11 compounds during physical exercise [28] | lipids | HemaPEN | UHPLC-MS/MS | Whole blood | 2023 |
| Monitoring of tacrolimus, creatinine and haemoglobin in kidney transplant recipients [29] | Tacrolimus, Hb and Creatinine | Mitra® device, Capitainer® B10 | LC-MS/MS | Whole blood | 2023 |
| Quantification of phosphatidylethanol (PEth) concentrations [30] | phosphatidylethanol (PEth) | Tasso-M20 | LC-MS/MS | Whole blood | 2023 |
| Quantitation of phenylalanine and tyrosine [31] | phenylalanine and tyrosine | Capitainer® B10 | Flow injection analysis tandem mass spectrometry (FIA-MS/MS) | Whole blood | 2023 |
| Neuro-ophthalmologic and blood biomarker responses in ADHD following subconcussive head impacts [32] | Neuro-ophthalmologic and blood biomarker | Tasso-M20 | Human Neurology 4-Plex A assay (N4PA) | Whole blood | 2023 |
| Stability of inflammation markers in human blood collected using volumetric absorptive microsampling (VAMS) under typical laboratory storage temperatures [33] | IL-6, IL-8 | Mitra® device | Luminex xMAP | Whole blood | 2023 |
| Patient-Centric Quantitative Microsampling for Accurate Determination of Urine Albumin to Creatinine Ratio (UACR) [34] | Urine Albumin to Creatinine Ratio | Mitra® device,  Capitainer® B10 | LC-MS/MS | Whole blood | 2023 |
| Examination of bile acid profiles [35] | Bile acid markers | Capitainer® B10 | LC-MS/MS | Feces | 2024 |
| Pharmacodynamic biomarker analysis [36] |  | Mitra® device, Tasso-M20, TassoOne plus. | Gyrolab immunoassay | Whole blood | 2024 |
| Detection of 26 Drugs of Abuse and Metabolites [37] | Metabolites | Capitainer® B10 | LC-MS/MS | Whole blood | 2024 |
| Proteome profiling of proteins involved in SARS-CoV-2 infections [38] | SARS-Cov-2 proteins | Capitainer® B10 | Affinity proteomics assays | Whole blood | 2024 |
| Monitoring of 25 Per- and polyfluoroalkyl substances (PFAS) [39] | Per- and polyfluoroalkyl substances (PFAS) | Capitainer® B10 | UHPLC-MS/MS | Whole blood | 2024 |
| Detection of Recombinant human erythropoietin (rhEPO) [40] | Recombinant human erythropoietin | Mitra® device, Capitainer® B50 | Western blot,  Erythropoietin (EPO) analysis,  Sarcosyl-Polyacrylamide gel electrophoresis (SAR-PAGE) | Whole blood, Urine | 2024 |
| Monitoring of Infliximab, Vedolizumab, and C-Reactive Protein Concentrations in Patients with Inflammatory Bowel Disease [41] | Infliximab, Vedolizumab, and C-RP | MiniCollect tubes | Enzyme-linked immunosorbent assay (ELISA) | Whole blood | 2024 |
| Analysis of small extracellular vesicles from dried blood spots [42] | Exosomes | “whole cells blood card” (ArrayIt Incorporation, CA, USA) | Western Blotting, Nanoparticle Tracking Analysis (NTA) | Whole blood | 2025 |
| Determination of Ustekinumab in Patients with Inflammatory Bowel Disease [43] | mAB | Whatman™ 903 Protein Saver Cards | ELISA | Whole blood | 2025 |
| Comparing the Transcriptomic Profile of Induced Immune Response via Lipopolysaccharide Stimulation in homeRNA and Venous Blood [44] | IL-6, IL-8, TNF-alfa | Tasso-SST | Luminex® Multiplex Immunoassays, ELISA | Whole blood | 2026 |

**References.**

1. Andriguetti, N. B., Lisboa, L. L., Hahn, S. R., Pagnussat, L. R., Antunes, M. V., & Linden, R. (2019). Simultaneous determination of vancomycin and creatinine in plasma applied to volumetric absorptive microsampling devices using liquid chromatography-tandem mass spectrometry. *Journal of Pharmaceutical and Biomedical Analysis*, *165*, 315–324. https://doi.org/10.1016/j.jpba.2018.12.023
2. Anoshkina, Y., Costas-Rodríguez, M., & Vanhaecke, F. (2017). Iron isotopic analysis of finger-prick and venous blood by multi-collector inductively coupled plasma-mass spectrometry after volumetric absorptive microsampling. *Journal of Analytical Atomic Spectrometry*, *32*(2), 314–321. https://doi.org/10.1039/C6JA00394J
3. Bæk, R., Sloth, J. K., Hasan, M. M., Midekessa, G., & Jørgensen, M. M. (2025). Analysis of small extracellular vesicles from dried blood spots. *Frontiers in Medical Technology*, *7*. https://doi.org/10.3389/fmedt.2025.1494239
4. Balduyck, M., Chapuis Cellier, C., Roche, D., Odou, M.-F., Joly, P., Madelain, V., Vergne, A., Nouadje, G., Lafitte, J.-J., Porchet, N., Beaune, P., & Zerimech, F. (2014). Development of a laboratory test on dried blood spots for facilitating early diagnosis of alpha-1-antitrypsin deficiency. *Annales de Biologie Clinique*, *72*(6), 689–704. https://doi.org/10.1684/abc.2014.1004
5. Beck, O., Mellring, M., Löwbeer, C., Seferaj, S., & Helander, A. (2021). Measurement of the alcohol biomarker phosphatidylethanol (PEth) in dried blood spots and venous blood—importance of inhibition of post-sampling formation from ethanol. *Analytical and Bioanalytical Chemistry*, *413*(22), 5601–5606. https://doi.org/10.1007/s00216-021-03211-z
6. Boutin, M., Lavoie, P., Beaudon, M., Kabala Ntumba, G., Bichet, D. G., Maranda, B., & Auray-Blais, C. (2023). Mass Spectrometry Analysis of Globotriaosylsphingosine and Its Analogues in Dried Blood Spots. *International Journal of Molecular Sciences*, *24*(4), 3223. https://doi.org/10.3390/ijms24043223
7. Brandsma, J., Chenoweth, J. G., Gregory, M. K., Krishnan, S., Blair, P. W., Striegel, D. A., Mehta, R., Schully, K. L., Dumler, J. S., Sikorski, C. C. S., O’Connor, K., Reichert-Scrivner, S. A., Paguirigan, C. M., Uyehara, C. F. T., Ngauy, C. V., Myers, C. A., & Clark, D. V. (2022). Assessing the use of a micro-sampling device for measuring blood protein levels in healthy subjects and COVID-19 patients. *PLOS ONE*, *17*(8), e0272572. https://doi.org/10.1371/journal.pone.0272572
8. Brown, L. G., Wei, X., Milton, L. A., Alizai, M. Y., MacDonald, J. W., Bammler, T. K., Zeng, Y., Robertson, I., Adams, K. N., Toh, Y.-C., Chaussabel, D., Berthier, E., Haack, A. J., & Theberge, A. B. (2026). From Home to Transcriptome: Comparing the Transcriptomic Profile of Induced Immune Response via Lipopolysaccharide Stimulation in homeRNA and Venous Blood. *Analytical Chemistry*, *98*(4), 2990–3001. https://doi.org/10.1021/acs.analchem.5c06040
9. Carling, R. S., Emmett, E. C., & Moat, S. J. (2022). Evaluation of volumetric blood collection devices for the measurement of phenylalanine and tyrosine to monitor patients with phenylketonuria. *Clinica Chimica Acta*, *535*, 157–166. https://doi.org/10.1016/j.cca.2022.08.005
10. Deprez, S., Van Uytfanghe, K., & Stove, C. P. (2023). Liquid chromatography-tandem mass spectrometry for therapeutic drug monitoring of immunosuppressants and creatinine from a single dried blood spot using the Capitainer® qDBS device. *Analytica Chimica Acta*, *1242*, 340797. https://doi.org/10.1016/j.aca.2023.340797
11. Engevik, M. A., Thapa, S., Lillie, I. M., Yacyshyn, M. B., Yacyshyn, B., Percy, A. J., Chace, D., & Horvath, T. D. (2024). Repurposing dried blood spot device technology to examine bile acid profiles in human dried fecal spot samples. *American Journal of Physiology-Gastrointestinal and Liver Physiology*, *326*(2), G95–G106. https://doi.org/10.1152/ajpgi.00188.2023
12. Fragala, M. S., Goldman, S. M., Goldman, M. M., Bi, C., Colletti, J. D., Arent, S. M., Walker, A. J., & Clarke, N. J. (2018). Measurement of Cortisol and Testosterone in Athletes: Accuracy of Liquid Chromatography–Tandem Mass Spectrometry Assays for Cortisol and Testosterone Measurement in Whole-Blood Microspecimens. *Journal of Strength and Conditioning Research*, *32*(9), 2425–2434. https://doi.org/10.1519/JSC.0000000000002726
13. Fredolini, C., Dodig-Crnković, T., Bendes, A., Dahl, L., Dale, M., Albrecht, V., Mattsson, C., Thomas, C. E., Torinsson Naluai, Å., Gisslen, M., Beck, O., Roxhed, N., & Schwenk, J. M. (2024). Proteome profiling of home-sampled dried blood spots reveals proteins of SARS-CoV-2 infections. *Communications Medicine*, *4*(1), 55. https://doi.org/10.1038/s43856-024-00480-4
14. Galletto, M., Ververi, C., Massano, M., Alladio, E., Vincenti, M., & Salomone, A. (2024). Development and validation of the UHPLC-MS/MS method for the quantitative determination of 25 PFAS in dried blood spots. *Analytical and Bioanalytical Chemistry*. https://doi.org/10.1007/s00216-024-05484-6
15. García-Poyo, M. C., Pécheyran, C., Rello, L., García-González, E., Alonso Rodríguez, S., Nakadi, F. V., Aramendía, M., & Resano, M. (2021). Determination of Cu in blood *via* direct analysis of dried blood spots using high-resolution continuum source graphite furnace atomic absorption spectrometry. *Journal of Analytical Atomic Spectrometry*, *36*(8), 1666–1677. https://doi.org/10.1039/D1JA00155H
16. Gunash, J., Aristizabal-Henao, J. J., & Stark, K. D. (2019). Quantitating fatty acids in dried blood spots on a common collection card versus a novel wicking sampling device. *Prostaglandins, Leukotrienes and Essential Fatty Acids*, *145*, 1–6. https://doi.org/10.1016/j.plefa.2019.05.002
17. Heiland, C. E., Lehtihet, M., Börjesson, A., & Ekström, L. (2024). Evaluation of a single Eporatio® micro‐dose in urine and dried blood spots. *Drug Testing and Analysis*. https://doi.org/10.1002/dta.3651
18. Jett, J. D., Beck, R., Tyutyunnyk, D., Sanchez, J., Lopez‐Cruzan, M., Ginsburg, B. C., McPherson, S. M., Javors, M. A., McDonell, M. G., & Hill‐Kapturczak, N. (2023). Validation of the quantification of phosphatidylethanol 16:0/18:1 concentrations in <scp>TASSO‐M20</scp> devices. *Alcohol: Clinical and Experimental Research*, *47*(4), 748–755. https://doi.org/10.1111/acer.15024
19. John, H., Willoh, S., Hörmann, P., Siegert, M., Vondran, A., & Thiermann, H. (2016). Procedures for Analysis of Dried Plasma Using Microsampling Devices to Detect Sulfur Mustard-Albumin Adducts for Verification of Poisoning. *Analytical Chemistry*, *88*(17), 8787–8794. https://doi.org/10.1021/acs.analchem.6b02199
20. Jones, C., Dunseath, G. J., Lemon, J., & Luzio, S. D. (2018). Microsampling Collection Methods for Measurement of C-peptide in Whole Blood. *Journal of Diabetes Science and Technology*, *12*(5), 1024–1028. https://doi.org/10.1177/1932296818763464
21. Joung, S., Dzubur, E., van den Broek, I., Love, A., Martinez-Rubio, L., Lopez, M., Noah, B., Dhawan, S., Fu, Q., Mastali, M., Van Eyk, J. E., Spiegel, B., Bairey Merz, C. N., & Shufelt, C. (2019). Early Detection of Atrial Fibrillation-Atrial Flutter Using Remote Patient Monitoring. *Journal of Medical Cases*, *10*(2), 31–36. https://doi.org/10.14740/jmc3151w
22. Laurent, A., Nix, C., Cobraiville, G., Crommen, J., & Fillet, M. (2023). A targeted UHPLC-MS/MS method to monitor lipidomic changes during a physical effort: Optimization and application to blood microsamples from athletes. *Journal of Pharmaceutical and Biomedical Analysis*, *229*, 115373. https://doi.org/10.1016/j.jpba.2023.115373
23. Li, W., Chace, D. H., & Garrett, T. J. (2023). Quantitation of phenylalanine and tyrosine from dried Blood/Plasma spots with impregnated stable isotope internal standards (SIIS) by FIA-SRM. *Clinica Chimica Acta*, *549*, 117551. https://doi.org/10.1016/j.cca.2023.117551
24. Lima, D. A., Schuch, R. A., Salgueiro, J. S., Pintão, M. C. T., & Carvalho, V. M. (2022). Evaluation of Volumetric Absorptive Microsampling and Mass Spectrometry Data-Independent Acquisition of Hemoglobin-Related Clinical Markers. *Journal of Proteome Research*, *21*(8), 1816–1828. https://doi.org/10.1021/acs.jproteome.1c00892
25. Löfgren, L., von Euler Chelpin, M., Bhat, M., Althage, M., Hober, A., Edfors, F., Ruckh, T., Challis, B., Davidsson, P., & Miliotis, T. (2024). Patient-Centric Quantitative Microsampling for Accurate Determination of Urine Albumin to Creatinine Ratio (UACR) in a Clinical Setting. *The Journal of Applied Laboratory Medicine*, *9*(2), 329–341. https://doi.org/10.1093/jalm/jfad111
26. Marasca, C., Arana, M. E. B., Protti, M., Cavalli, A., Mercolini, L., & Armirotti, A. (2021). Volumetric Absorptive Microsampling of Blood for Untargeted Lipidomics. *Molecules*, *26*(2), 262. https://doi.org/10.3390/molecules26020262
27. Marchand, A., Roulland, I., Semence, F., Beck, O., & Ericsson, M. (2021). Use of Quantitative Dried Blood Spots to Evaluate the Post-Vaccination Level of Neutralizing Antibodies against SARS-CoV-2. *Life*, *11*(11), 1125. https://doi.org/10.3390/life11111125
28. Marshall, D. J., Adaway, J. E., Hawley, J. M., & Keevil, B. G. (2020). Quantification of testosterone, androstenedione and 17-hydroxyprogesterone in whole blood collected using Mitra microsampling devices. *Annals of Clinical Biochemistry: International Journal of Laboratory Medicine*, *57*(5), 351–359. https://doi.org/10.1177/0004563220937735
29. Mathew, B. S., Mathew, S. K., Aruldhas, B. W., Prabha, R., Gangadharan, N., David, V. G., Varughese, S., & John, G. T. (2022). Analytical and clinical validation of dried blood spot and volumetric absorptive microsampling for measurement of tacrolimus and creatinine after renal transplantation. *Clinical Biochemistry*, *105–106*, 25–34. https://doi.org/10.1016/j.clinbiochem.2022.04.014
30. McMahon, R., Hill, C., Rudge, J., Herbert, B., & Karsten, E. (2023). Stability of inflammation markers in human blood collected using volumetric absorptive microsampling (VAMS) under typical laboratory storage temperatures. *Cytokine*, *171*, 156355. https://doi.org/10.1016/j.cyto.2023.156355
31. Meikopoulos, T., Begou, O., Theodoridis, G., & Gika, H. (2023). Ceramides biomarkers determination in quantitative dried blood spots by UHPLC-MS/MS. *Analytica Chimica Acta*, *1255*, 341131. https://doi.org/10.1016/j.aca.2023.341131
32. Meikopoulos, T., Gika, H., Theodoridis, G., & Begou, O. (2024). Detection of 26 Drugs of Abuse and Metabolites in Quantitative Dried Blood Spots by Liquid Chromatography–Mass Spectrometry. *Molecules*, *29*(5), 975. https://doi.org/10.3390/molecules29050975
33. Mingas, P.-D., Aguiar Zdovc, J., Grabnar, I., Drobne, D., & Vovk, T. (2025). Development, Validation and Application of the Dried Blood Spot Analysis Method for the Determination of Ustekinumab in Patients with Inflammatory Bowel Disease. *Pharmaceuticals*, *18*(9), 1253. https://doi.org/10.3390/ph18091253
34. Mongongu, C., Moussa, E. M., Semence, F., Roulland, I., Ericsson, M., Coudoré, F., Marchand, A., & Buisson, C. (2020). Use of Capillary Dried Blood for Quantification of Intact IGF-I by LC–HRMS for Antidoping Analysis. *Bioanalysis*, *12*(11), 737–752. https://doi.org/10.4155/bio-2020-0013
35. Nakadi, F. V., Garde, R., da Veiga, M. A. M. S., Cruces, J., & Resano, M. (2020). A simple and direct atomic absorption spectrometry method for the direct determination of Hg in dried blood spots and dried urine spots prepared using various microsampling devices. *Journal of Analytical Atomic Spectrometry*, *35*(1), 136–144. https://doi.org/10.1039/C9JA00348G
36. Nix, C., Hemmati, M., Cobraiville, G., Servais, A.-C., & Fillet, M. (2021). Blood Microsampling to Monitor Metabolic Profiles During Physical Exercise. *Frontiers in Molecular Biosciences*, *8*. https://doi.org/10.3389/fmolb.2021.681400
37. Nowak, M. K., Kronenberger, W. G., Rettke, D., Ogbeide, O., Klemsz, L. M., Quinn, P. D., Mickleborough, T. D., Newman, S. D., & Kawata, K. (2023). Neuro-ophthalmologic and blood biomarker responses in ADHD following subconcussive head impacts: a case–control trial. *Frontiers in Psychiatry*, *14*. https://doi.org/10.3389/fpsyt.2023.1230463
38. Nys, G., Gallez, A., Kok, M. G. M., Cobraiville, G., Servais, A.-C., Piel, G., Pequeux, C., & Fillet, M. (2017). Whole blood microsampling for the quantitation of estetrol without derivatization by liquid chromatography-tandem mass spectrometry. *Journal of Pharmaceutical and Biomedical Analysis*, *140*, 258–265. https://doi.org/10.1016/j.jpba.2017.02.060
39. Otten, A. T., van der Meulen, H. H., Steenhuis, M., Loeff, F. C., Touw, D. J., Kosterink, J. G. W., Frijlink, H. W., Rispens, T., Dijkstra, G., Visschedijk, M. C., & Bourgonje, A. R. (2023). Clinical Validation of a Capillary Blood Home-Based Self-Sampling Technique for Monitoring of Infliximab, Vedolizumab, and C-Reactive Protein Concentrations in Patients With Inflammatory Bowel Disease. *Inflammatory Bowel Diseases*. https://doi.org/10.1093/ibd/izad103
40. Protti, M., Cirrincione, M., Mandrioli, R., Rudge, J., Regazzoni, L., Valsecchi, V., Volpi, C., & Mercolini, L. (2022). Volumetric Absorptive Microsampling (VAMS) for Targeted LC-MS/MS Determination of Tryptophan-Related Biomarkers. *Molecules*, *27*(17), 5652. https://doi.org/10.3390/molecules27175652
41. Verougstraete, N., Stove, V., & Stove, C. (2018). Wet absorptive microsampling at home for HbA1c monitoring in diabetic children. *Clinical Chemistry and Laboratory Medicine (CCLM)*, *56*(12), e291–e294. https://doi.org/10.1515/cclm-2018-0207
42. Vethe, N. T., Åsberg, A., Andersen, A. M., Heier Skauby, R., Bergan, S., & Midtvedt, K. (2023). Clinical performance of volumetric finger‐prick sampling for the monitoring of tacrolimus, creatinine and haemoglobin in kidney transplant recipients. *British Journal of Clinical Pharmacology*, *89*(12), 3690–3701. https://doi.org/10.1111/bcp.15870
43. Wang, J., Li, D., Wiltse, A., Emo, J., Hilchey, S. P., & Zand, M. S. (2019). Application of volumetric absorptive microsampling (VAMS) to measure multidimensional anti-influenza IgG antibodies by the mPlex-Flu assay. *Journal of Clinical and Translational Science*, *3*(6), 332–343. https://doi.org/10.1017/cts.2019.410
44. Yang, X., Logis, E., Williams, K., Sheng, X. R., & Fischer, S. K. (2024). Evaluation of low volume sampling devices for a pharmacodynamic biomarker analysis: Challenges and solutions. *Journal of Pharmaceutical and Biomedical Analysis*, *251*, 116454. https://doi.org/10.1016/j.jpba.2024.116454
